# Gut-Derived Lipid Mediators Orchestrate Ovarian Metabolic Homeostasis and Clutch Persistence in Aging Laying Hens via the PLA2G6-ALOX15B-AGPAT3 Axis

**DOI:** 10.3390/biom16050708

**Published:** 2026-05-11

**Authors:** Xin Li, Xiaoliang Wang, Xia Cai, Qiang Meng, Yanyan Sun, Changsuo Yang, Junfeng Yao

**Affiliations:** 1 Institute of Animal Husbandry and Veterinary Science, Shanghai Academy of Agricultural Sciences, Shanghai 201106, China; lixinxinli_sadu@outlook.com (X.L.); wxlyjf@163.com (X.W.); acandaf@163.com (X.C.); 15525630527@163.com (Q.M.); yangchangsuo@189.cn (C.Y.); 2 Institute of Animal Science, Chinese Academy of Agricultural Sciences, Beijing 100193, China; yanyansun2014@163.com

**Keywords:** gut–ovary axis, clutch persistence, lipid mediators, PLA2G6-ALOX15B-AGPAT3 axis, laying hens

## Abstract

Clutch persistence, defined as the ability to sustain consecutive egg-laying cycles, is a pivotal determinant of profitability in the poultry industry, particularly for aging laying hens (≥65 weeks). However, the molecular mechanisms governing this trait remain elusive, largely due to the traditional “ovary-centric” paradigm that overlooks systemic regulation by the gut microbiota. To address this knowledge gap, the present study aimed to dissect the comprehensive regulatory network governing clutch persistence using integrated multi-omics analyses. A total of 20 sixty-five-week-old Rhode Island Red (RIR) laying hens with cumulative egg production exceeding 300 eggs but distinct clutch persistence were stratified into a high-clutch persistence group (HCP, ≥25 clutches, *n* = 10) and a low-clutch persistence group (LCPLCP, ≤15 clutches, *n* = 10). Multi-omics profiling, including ovarian transcriptomics, proteomics, and metabolomics; serum metabolomics; and cecal microbiota 16S rRNA sequencing was performed. Data integration and association mining were conducted via Spearman correlation analysis with stringent thresholds (r > 0.6, *p* < 0.01). Integrated analyses revealed a “gut–ovary axis” regulatory model mediated by a lipid mediator network, operating through a three-tiered mechanism: (1) Gut Initiation: The HCP group exhibited enriched cecal γ-Proteobacteria, which promoted biosynthesis of lipid precursors. (2) Serum Transport: Key serum lipid mediators, most notably LysoPC (22:6) (VIP = 4.5) and cholesterol ester CE (20:4), served as critical carriers transducing gut-derived signals to the ovary. (3) Ovarian Execution: These lipid signals activated a core ovarian metabolic pathway centered on the PLA2G6-ALOX15B-AGPAT3 axis, which coordinated follicular development and ovulation by supplying steroid hormone synthesis substrates, exerting anti-inflammatory effects, and stabilizing membrane structures. Collectively, this study demonstrates that gut microbiota modulates clutch persistence in aging laying hens via lipid mediators, orchestrating a systemic “gut–serum–ovary” regulatory cascade. These findings provide a novel molecular framework for extending the economic egg-laying cycle through the targeted manipulation of intestinal microbiota or serum lipid metabolism.

## 1. Introduction

Laying performance is a core economic indicator of the poultry industry, while clutch egg-laying continuity (the ability to maintain a continuous egg-laying cycle) exerts a particularly prominent impact on the production efficiency of aged laying hens (≥65 weeks of age) [1]. Although genetic breeding has significantly improved the total lifetime egg production of laying hens (reaching 500 eggs by 100 weeks of age) [2], the sustainability of egg production in the advanced age stage remains a key bottleneck restricting production profitability. Clutch egg-laying continuity is quantified by “egg-laying clusters”: consecutive laying of ≥2 eggs defines one cluster, and a new cycle initiates with an interval of ≥1 day. High-yield and persistent individuals optimize feed utilization efficiency by reducing non-production days; in contrast, even with equivalent total egg production, individuals with low continuity exhibit diminished economic benefits due to their dispersed laying rhythm. This is a phenomenon particularly pronounced in hens ≥65 weeks of age and those with an annual egg production exceeding 300 eggs. Less than 10% of these hens demonstrate high laying sustainability, highlighting the practical significance of elucidating regulatory mechanisms. Egg-laying cluster size, as an inherent trait, serves as a valuable breeding marker [3,4,5,6], yet the complex physiological and molecular drivers governing this trait remain incompletely understood.

The continuity of egg laying is fundamentally orchestrated by the hypothalamic–pituitary–gonadal (HPG) axis, which governs the hierarchical development of ovarian follicles. In hens, a strict follicular hierarchy (F1–F5) is maintained through the precise pulsatile secretion of gonadotropin-releasing hormone (GnRH), triggering pre-ovulatory surges of luteinizing hormone (LH) and follicle-stimulating hormone (FSH) [7,8]. The selection of follicles for rapid growth depends on cellular sensitivity to these gonadotropins [9,10], while the termination of a clutch is often linked to desynchronization between the terminal follicle (Ct) and the recruitment of the next follicle (C1) [11,12]. Crucially, this process is heavily dependent on lipid metabolism, as the liver-derived very-low-density lipoproteins (VLDLs) and vitellogenin serve as essential precursors for yolk formation and steroid hormone synthesis [13,14]. Disruptions in lipid signaling can trigger follicular atresia, breaking the clutch sequence [15]. Thus, sustained egg production requires a seamless integration of neuroendocrine signals and metabolic support.

However, traditional research has largely adopted an “ovary-centric” paradigm, focusing on local ovarian mechanisms while overlooking systemic, long-range regulatory factors. Emerging evidence suggests that the gut microbiota acts as a virtual endocrine organ, modulating host metabolism via metabolites like short-chain fatty acids and lipid mediators [16,17]. While a “gut–ovary axis” has been proposed in mammals [18,19], it remains unknown whether and how specific cecal microbial communities in aged laying hens drive clutch persistence by reshaping the serum lipid landscape and subsequently regulating ovarian gene networks. Addressing this gap is not merely an academic exercise; it is essential for shifting the industry’s focus from simple genetic selection to holistic metabolic management.

To address this challenge, we present the first systematic dissection of the ‘gut–serum–ovary’ regulatory axis in aged laying hens exhibiting distinct clutch persistence phenotypes. By integrating ovarian transcriptomics, proteomics, and metabolomics with serum metabolomics and cecal microbiome profiling, our study moves beyond simple correlations to identify specific microbial taxa and lipid mediators that functionally link gut dysbiosis to ovarian follicular fate. This multi-omics approach elucidates how gut-derived signals transduce regulatory cues to modulate HPG axis efficiency and local lipid metabolism, thereby establishing a novel theoretical framework for reproductive regulation. Ultimately, by pinpointing actionable targets such as specific probiotics or lipid supplements, these findings provide a scientific basis for developing nutritional strategies to extend the economic egg-laying cycle beyond 72 weeks, enhancing both the sustainability and profitability of the poultry industry [20,21].

## 2. Materials and Methods

### 2.1. Ethical Statement

All experimental procedures in this study were approved by the Animal Welfare Committee of Shanghai Academy of Agricultural Sciences (Approval Number: SAASPZ0523078). To minimize the stress in laying hens, the laying hens were fasted for 12 h and then euthanized by cervical dislocation.

### 2.2. Animal Selection and Phenotyping

A total of 3000 individually caged 65-week-old Rhode Island Red hens constituted the initial experimental population, with individual egg production continuously recorded by an automatic egg collection system. Hens with a cumulative egg production of over 300 eggs from 20 to 65 weeks of age were included for phenotypic assessment. An egg-laying cluster was defined as continuous oviposition of no fewer than two eggs, while a laying interval of at least one day indicated the start of a new cluster cycle.

To obtain distinct phenotypic differences in clutch persistence, an extreme grouping strategy was applied. Hens ranking in the top 10% (≥25 clusters) were assigned to the HCP group, and those in the bottom 10% (≤15 clusters) were defined as the LCP group, with ten biological replicates in each group. A two-phase sampling design was adopted for multi-omics analysis. Within each group, three individuals with clutch numbers closest to the group mean were selected for ovarian transcriptome sequencing, and five representative hens were used for targeted serum metabolomic detection, balancing biological representativeness and experimental feasibility.

### 2.3. Diet and Housing

All hens were housed in the same environmentally controlled facility under a 16L:8D photoperiod. The ambient temperature was maintained at 22 ± 1 °C, and relative humidity was controlled at 60–70%. Hens were kept in individual cages and had *ad libitum* access to feed and water via an automated feeding and drinking system throughout the experiment.

To eliminate diet composition as a confounding variable, all hens were fed the same batch of a standard corn–soybean meal-based layer diet formulated to meet or exceed the nutrient requirements for laying hens. The detailed composition and calculated nutritional levels of the diet are provided in Table 1.

### 2.4. Sample Collection

Within 5 min post-euthanasia, the ovaries were completely dissected and randomly cut into three ~0.5 g fragments for different pretreatment procedures. Transcriptome samples were fixed in RNA-later (4 °C, 24 h) and stored at −80 °C. Proteomic samples were homogenized on ice in RIPA lysis buffer supplemented with protease inhibitors and stored at −80 °C. Metabolome samples were immediately immersed in pre-cooled methanol: water (80:20, *v*/*v*), vortexed to inactivate enzyme activity, and stored at −80 °C. All ovarian tissue manipulations were performed on ice within 10 min to avoid cross-contamination.

Blood samples were collected via the wing vein and centrifuged at 3500 rpm for 10 min at 4 °C to separate serum. The resulting serum from each individual was equally divided into two aliquots on ice within 5 min (volume error <2%)—one for targeted metabolomics and the other for non-targeted metabolomics analysis. For non-targeted metabolomics, serum was precipitated with acetonitrile (1:4, *v*/*v*), and the supernatant was freeze-dried and reconstituted in an appropriate LC–MS-compatible solvent. Samples were analyzed using LC–QTOF/MS in both positive and negative ionization modes. Quality control (QC) samples, prepared by pooling equal volumes of all serum samples, were injected regularly throughout the analytical batch to ensure data stability and reproducibility. For targeted metabolomics, 40 μL of serum was spiked with internal standards (Avanti Polar Lipids, Alabaster, AL, USA) and subjected to liquid–liquid extraction using methyl tert-butyl ether and methanol (5:1, *v*/*v*). The extract was dried under vacuum at 37 °C and reconstituted in a solvent mixture of dichloromethane, methanol, and water (60:30:4.5, *v*/*v*/*v*). After centrifugation (12,000 rpm, 15 min, and 4 °C), the supernatant was analyzed by UHPLC–MS/MS in multiple reaction monitoring (MRM) mode.

Cecal contents were aseptically collected and homogenized in PBS buffer. A 0.5 g aliquot was subjected to 16S rRNA gene sequencing, with DNA extracted using the MOBIO PowerSoil Kit(MO BIO Laboratories, Carlsbad, CA, USA).

### 2.5. Multi-Omics Profiling

#### 2.5.1. Cecal Microbiome

Total DNA was extracted from cecal contents using the E.Z.N.A.^®^ Soil DNA Kit(Omega Bio-tek, Norcross, GA, USA). After quality verification via 1% agarose gel electrophoresis and NanoDrop2000, the V3-V4 region of the 16S rRNA gene was amplified with 338F (5′-ACTCCTACGGGAGGCAGCAG-3′)/806R(5′-GGACTACHVGGGTWTCTAAT-3′) primers. The PCR program was as follows: pre-denaturation at 95 °C for 3 min, followed by 27 cycles of denaturation at 95 °C for 30 s, annealing at 55 °C for 30 s, and extension at 72 °C for 30 s. PCR products were recovered and purified by 2% agarose gel electrophoresis, and then paired-end sequenced on the Illumina Nextseq2000 platform. Raw data was processed with fastp (filtering bases with Q-value < Q20, sequences < 50 bp in length, and those containing ambiguous “N” bases) and FLASH (minimum overlap of 10 bp, maximum mismatch rate of 0.2). The ASV (Amplicon Sequence Variant) feature table was generated using the DADA2 plugin in QIIME2. After excluding chloroplast and mitochondrial sequences, samples were rarefied to 20,000 sequences (Good’s coverage > 99%), and taxonomic annotation was performed against the SILVA database (v138). Subsequent analyses were conducted on the Meiji Cloud platform: Alpha diversity indices (Chao1 and Shannon) were calculated with intergroup differences tested by the Wilcoxon rank-sum test; principal coordinate analysis (PCoA) based on Bray–Curtis distance was performed to assess community structure differences (PERMANOVA test), and differentially abundant microbiota were identified via LEfSe with thresholds of LDA score > 2 and *p* < 0.05.

#### 2.5.2. Ovarian/Serum Metabolome

Frozen ovarian tissue was ground to a fine powder in liquid nitrogen, followed by metabolite extraction with pre-chilled methanol/water (4:1, *v*/*v*) containing L-2-chlorophenylalanine (Sigma-Aldrich, St. Louis, MO, USA) as an internal standard. The homogenate underwent cryogenic grinding and low-temperature ultrasonication, then centrifuged (12,000 rpm, 15 min, and 4 °C). The supernatant was collected for non-targeted analysis.

Serum proteins were precipitated by adding cold acetonitrile/methanol (1:1, *v*/*v*), vortexed, and incubated at –20 °C for 30 min. After centrifugation, the supernatant was dried under a gentle nitrogen stream and reconstituted in acetonitrile/water (1:1, *v*/*v*).

A separate serum aliquot was processed using liquid–liquid extraction with methyl tert-butyl ether (MTBE) and methanol (5:1, *v*/*v*) spiked with isotope-labeled internal standards (Avanti Polar Lipids, Alabaster, AL, USA). The organic phase was evaporated and reconstituted in dichloromethane/methanol/water (60:30:4.5, *v*/*v*/*v*) prior to analysis.

For both non-targeted and targeted workflows, quality control (QC) samples were prepared by pooling equal volumes of all experimental samples. One QC sample was injected after every 5–15 analytical runs to monitor system stability.

Non-targeted metabolomic profiling was performed on a UHPLC-Q Exactive HF-X mass spectrometer coupled with an HSS T3 column (100 × 2.1 mm, 1.8 μm). The mobile phases were: (A) 0.1% formic acid in water/acetonitrile (95:5, *v*/*v*) and (B) acetonitrile/isopropanol/water (47.5:47.5:5, *v*/*v*/*v*). Data were acquired in both positive and negative ionization modes with an *m*/*z* range of 70–1050, with MS^1^ resolution of 60,000 and MS^2^ resolution of 7500.

Targeted metabolomics was conducted on a UHPLC–triple quadrupole MS system operating in multiple reaction monitoring (MRM) mode, with optimized transitions for each lipid mediator.

Raw non-targeted data were processed using Progenesis QI v3.0 (Waters Corporation, Milford, MA, USA) software for peak detection, alignment, and metabolite annotation against HMDB, METLIN, and in-house libraries. All data were uploaded to the Meiji Cloud platform for preprocessing: features with >20% missing values in any group were removed; remaining missing values were imputed with the minimum observed value; and total sum normalization and log_10_ transformation were applied. Additionally, metabolites with relative standard deviation (RSD) > 30% in QC samples were excluded. Differentially expressed metabolites (DEMs) were identified via OPLS-DA (VIP > 1 and *p* < 0.05) and subjected to KEGG pathway enrichment analysis.

To ensure high confidence in metabolite identification and eliminate false positives, we implemented a strict multi-step manual validation protocol:

Only annotations with an MS/MS spectral similarity score ≥ 30 (out of 100) were retained. Features with zero or low-confidence fragmentation matches were discarded, regardless of precursor mass accuracy.

All putative identifications were cross-referenced with the experimental conditions. Annotations corresponding to exogenous pharmaceuticals, synthetic chelators, or specific mycotoxins that were not administered to the animals or present in the certified feed were rigorously excluded to prevent misidentification of isobaric endogenous interferences.

Retention times were evaluated for consistency with the predicted polarity and physicochemical properties of the annotated compounds within our reverse-phase chromatography system. Features with elution profiles inconsistent with their chemical nature were flagged and removed.

The metabolite identification process included a confidence level assignment: compounds were classified as Level 2 (putatively annotated) based on diagnostic MS/MS spectra, or as Level 1 (confirmed identification) when authentic chemical standards were available for verification. Putative annotations lacking sufficient spectral evidence (Level 3) were excluded from biological interpretation.

Following this rigorous filtering and validation process, differentially expressed metabolites (DEMs) were identified via OPLS-DA (VIP > 1 and *p* < 0.05) and subjected to KEGG pathway enrichment analysis.

#### 2.5.3. Ovarian Transcriptome

Ovarian tissue was ground in liquid nitrogen, and total RNA was extracted using QIAzol Lysis Reagent (Qiagen, Hilden, Germany). RNA purity was determined using a NanoDrop2000 (OD260/280 = 1.8–2.2), and integrity was verified via an Agilent 5300 (RNA Qualified Number, RQN > 6.5). Qualified samples (RNA concentration ≥ 30 ng/μL) were used for library construction with the Illumina Stranded mRNA Prep kit: mRNA was enriched using Oligo (dT) magnetic beads, fragmented to ~300 bp, and reverse-transcribed into double-stranded cDNA. After end repair and adapter ligation, 300–400 bp fragments were selected for PCR amplification. Libraries were quantified using a Qubit 4.0 and sequenced on the NovaSeq X Plus platform with 150 bp paired-end reads (≥40 million reads/samples). Bioinformatics analysis was performed as follows: ① Low-quality reads were filtered using fastp (v0.23.2); ② Clean reads were aligned to the bGalGal1. Mat. Broiler. GRCg7b genome using HISAT2 (v2.2.1); ③ Transcripts per million (TPM) values were calculated using RSEM (v1.3.3); ④ Differentially expressed genes (DEGs) were identified using DESeq2 (v1.38.3) with the criteria of |log_2_ fold change (FC)| > 1 and false discovery rate (FDR) < 0.05; ⑤ Gene Ontology (GO) and Kyoto Encyclopedia of Genes and Genomes (KEGG) enrichment analyses were conducted using Goatools (v1.1.6) and SciPy (v1.10.0), respectively, with statistical significance defined as Bonferroni-corrected *p* < 0.05.

#### 2.5.4. Ovarian Proteome

Frozen ovarian tissue was homogenized in lysis buffer containing protease inhibitors (8 M urea, 1% SDS) using a tissue grinder (180 s × 3 cycles) and subsequently sonicated on ice for 30 min. The lysate was centrifuged, and the supernatant was collected for protein quantification using the BCA assay. A 100 μg aliquot of protein was dissolved in 100 mM triethylammonium bicarbonate (TEAB). Reduction was performed by adding 10 mM tris (2-carboxyethyl)phosphine (TCEP) and incubating at 37 °C for 60 min, followed by alkylation with 40 mM iodoacetamide at room temperature for 40 min in the dark. Proteins were then precipitated with cold acetone, reconstituted, and digested overnight with trypsin at 37 °C. The resulting peptides were desalted, concentrated, and quantified using a NanoDrop spectrophotometer (Thermo Fisher Scientific, Waltham, MA, USA). Chromatographic separation was carried out on a Vanquish Neo UHPLC system equipped with a uPAC High-Throughput column (75 μm × 5.5 cm) using an 8 min gradient elution with mobile phases consisting of 0.1% formic acid in water (A) and acetonitrile (B). Peptides were analyzed on an Orbitrap Astral mass spectrometer (Thermo Fisher Scientific, Waltham, MA, USA) operated in data-independent acquisition (DIA) mode with positive ionization and a spray voltage of 1.5 kV, and a scan range of *m*/*z* 100–1700. Raw data were processed using Spectronaut 19 software to identify differentially expressed proteins (DEPs), defined as those with |log_2_(fold change)| ≥ 0.263 and *p* < 0.05. Functional enrichment analysis (GO and KEGG pathways) and protein–protein interaction (PPI) network construction were performed on the Meiji Cloud Platform.

### 2.6. Multi-Omics Data Integration Analysis

Multi-omics association mining was performed on the Meiji Cloud platform, integrating proteomic, metabolomic, and cecal microbiome data. First, Spearman correlation analysis was conducted between differentially expressed proteins (DEPs, |log_2_ fold change (FC)| ≥ 0.263, *p* < 0.05), differentially expressed metabolites (DEMs, VIP > 1, *p* < 0.05), and differentially abundant bacterial genera (LDA score >2, *p* < 0.05) with thresholds of correlation coefficient r > 0.6 and *p* < 0.01, to construct a protein–metabolite–microbe interaction network. Second, joint KEGG pathway annotation was performed to map the three-omics data to shared metabolic pathways, enabling identification of cross-omics regulatory nodes. Finally, distance-based redundancy analysis (db-RDA) was used to quantify the association strength between microbial community structure and host proteins/metabolites, thereby revealing the “microbiota–host” co-metabolic mechanism. All analyses were completed on the Meiji Cloud platform, with data interaction visualization implemented via its built-in multi-omics integration module.

### 2.7. Bioinformatic Analysis of Differentially Expressed Proteins

To systematically characterize the physicochemical properties, structural features, and functional roles of DEPs identified in proteomic profiling, integrated bioinformatic analyses were performed, including hydrophobicity assessment, three-dimensional (3D) structure prediction, and subcellular localization annotation. These analyses were conducted using publicly available databases and computational tools, ensuring reliable validation of DEP functions associated with lipid mediator networks and clutch egg-laying persistence mechanisms.

#### 2.7.1. Hydrophobicity Estimation

Hydrophobicity scores of DEPs were calculated using the ProtParam tool (ExPASy; https://web.expasy.org/protparam/, accessed on 6 February 2026) based on the Kyte–Doolittle scale. This method quantifies the relative hydrophobicity of amino acid sequences, with scores > 0 indicating hydrophobic regions and scores < 0 indicating hydrophilic domains. Input sequences for DEPs were derived from UniProtKB (https://www.uniprot.org/, accessed on 6 February 2026). A sliding window size of 9 amino acids was used to smooth local fluctuations, and results were normalized to a scale of −2 to +2. Hydrophobicity profiles were employed to infer membrane association potential and lipid-binding capacity, which aligns with the lipid metabolism pathways discussed in subsequent results.

#### 2.7.2. Three-Dimensional Structure Prediction

Three-dimensional (3D) structures of DEPs were predicted using AlphaFold v2.0, with default parameters for monomeric protein folding. Input sequences were retrieved from the NCBI RefSeq database. AlphaFold generated five models per protein, and the highest-confidence model was selected based on a predicted Local Distance Difference Test (pLDDT) score > 70, indicating high structural reliability. Structural validation was performed via SWISS-MODEL (https://swissmodel.expasy.org/, accessed on 6 February 2026) using template-based homology modeling, with a sequence identity threshold of >30% to ensure reliable alignment. Predicted structures were visualized and analyzed for functional domains using PyMOL v2.5, with a focus on residues involved in lipid metabolism.

#### 2.7.3. Subcellular Localization Prediction

Subcellular localization of DEPs was predicted via a consensus approach integrating three tools:

WoLF PSORT (https://www.genscript.com/wolf-psort.html, accessed on 6 February 2026) for eukaryotic sequences, which analyzes amino acid composition, sorting signals, and functional motifs.

TargetP v2.0 for identifying N-terminal targeting peptides.

DeepLoc v1.0 for deep learning-based predictions using protein sequence embeddings.

Input sequences were annotated with UniProt IDs, and predictions were cross-validated against the COMPARTMENTS database (https://compartments.jensenlab.org/, accessed on 6 February 2026) to incorporate experimental evidence. High-confidence predictions were defined by WoLF PSORT (https://www.genscript.com/wolf-psort.html, accessed on 6 February 2026) scores > 10 and DeepLoc probability scores > 0.8. These results were used to infer DEP roles in organelle-specific lipid synthesis and signal transduction, directly supporting the subsequent ovarian pathway analyses.

### 2.8. qRT-PCR

Total RNA was isolated from ovarian tissues collected from HCP and LCP groups (*n* = 5 per group) using TRIzol^®^ reagent (Invitrogen, Carlsbad, CA, USA). The concentration and purity of the extracted RNA were determined using a NanoDrop 1000 UV spectrophotometer (Thermo Scientific, Waltham, MA, USA) by measuring the absorbance at 260/280 nm. Subsequently, 1.0 μg of total RNA was reverse transcribed into cDNA using the HiScript^®^ III RT SuperMix for qPCR (+gDNA wiper) kit (Vazyme Biotech Co. Ltd., Nanjing, China), strictly following the manufacturer’s instructions.

Quantitative real-time PCR (qRT-PCR) was performed using the ChamQTM Universal SYBR^®^ qPCR Master Mix (Vazyme Biotech Co. Ltd., Nanjing, China) on a CFX96 Touch Real-Time PCR Detection System (Bio-Rad, USA). The reaction system was set up as follows: 10.0 μL of 2× ChamQ Universal SYBR qPCR Master Mix, 0.4 μL of each primer (10 μM), 2.0 μL of cDNA template, and 7.2 μL of nuclease-free water, resulting in a final volume of 20.0 μL. The thermal cycling conditions consisted of an initial denaturation at 95 °C for 30 s, followed by 40 cycles of denaturation at 95 °C for 5 s and annealing/extension at 60 °C for 30 s. Melt curve analysis was conducted to confirm the specificity of the amplification products.

The relative mRNA expression levels of the target genes (*PLA2G6*, *ALOX15B*, and *AGPAT3*) were calculated using the 2−ΔΔCt method [22]. All data were normalized against the housekeeping gene *β-actin*. The sequences of the primers used for qRT-PCR are listed in Table 2. Statistical analysis was performed using Student’s t-test to compare the gene expression differences between the HCP and LCP groups, with *p* < 0.05 considered statistically significant.

## 3. Results

### 3.1. Results of Cecal Microbiota Diversity Analysis

#### 3.1.1. Principal Coordinate Analysis (PCoA)

**Figure 1:**
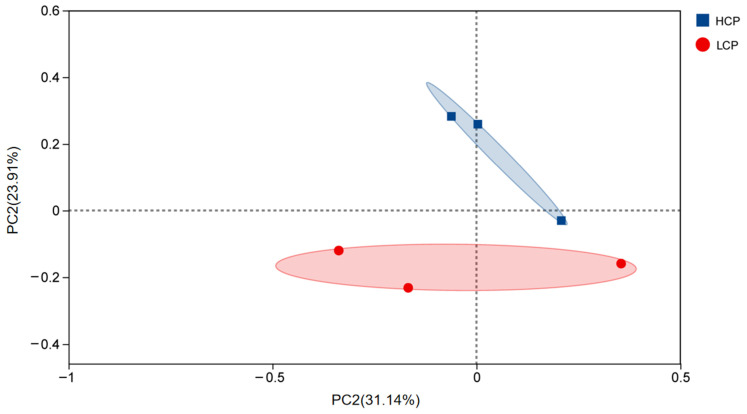
PCoA of cecal microbial communities at the ASV level and box plot of PC1 axis interpretation values among groups. Note: HCP group is represented by blue squares, and LCP group is represented by red circles. The blue and red shaded ellipses represent the 95% confidence intervals for each group. The interpretation values of the PC1 axis across different groups are presented in a box plot to facilitate the intuitive comparison of inter-group differences.

PCoA based on amplicon sequence variant (ASV) levels revealed significant differences in cecal microbiota structure between the HCP (blue squares) and the LCP (red dots) groups (Figure 1). The two-dimensional PCoA space was defined by PC1 (explaining 31.14% of total variation) and PC2 (explaining 23.91% of total variation); HCP samples were concentrated in the positive quadrants of both PC1 and PC2, while LCP samples aggregated distinctly in the negative regions of both axes, with no overlap in inter-group distribution boundaries. PC1 served as the dominant dimension driving grouping differentiation, with HCP samples localized in the PC1 positive zone and LCP samples in the PC1 negative zone. In contrast, PC2 primarily reflected intra-group variation, with HCP samples showing higher dispersion along the PC2 axis. The cumulative explanatory power of PC1 and PC2 reached 55.05%. Permutational multivariate analysis of variance (PERMANOVA) confirmed that clutch egg-laying persistence was the key driver of cecal microbiota β diversity differentiation (*p* = 0.002, R = 0.48, permutation number = 999). Notably, the LCP group exhibited higher homogeneity in microbiota structure (outlined by pink ellipses), whereas the dispersion of HCP samples along PC2 may be attributed to individual variations in functional bacteria abundance.

#### 3.1.2. Community Bar Plot and Pie Plot

**Figure 2:**
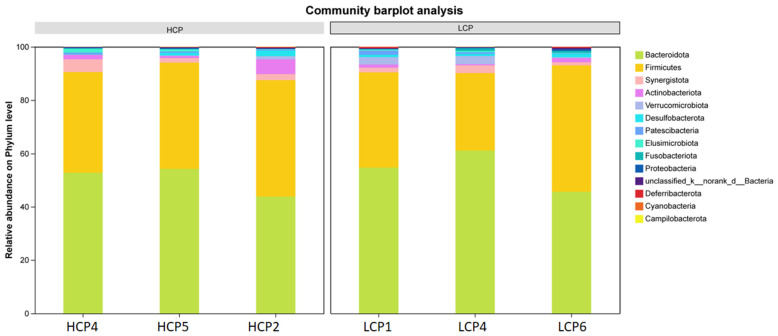
Bar plot and Pie plot of cecal microbiota community composition. Note: For the bar plot, the abscissa represents sample names, and the ordinate indicates the relative abundance of each microbial species in the corresponding sample. Columns of different colors correspond to different microbial species, with column length reflecting species abundance. Group labels are marked on the columns to distinguish the HCP and LCP groups.

The results via bar plot revealed significant differences in the relative abundance of intestinal microbiota at the phylum level between the HCP group and LCP group (Figure 2). In the HCP group, Bacteroidota (yellowish-green) was the dominant phylum, accounting for 50–70% of the total microbiota, followed by Firmicutes (yellow, 20–40%), while other phyla collectively contributed 5–10%. In contrast, the LCP group was dominated by Firmicutes (40–60%), with Bacteroidetes representing a significantly lower proportion (30–50%, *p* < 0.05), and all other phyla each comprising approximately 5%. Consequently, the Firmicutes-to-Bacteroidota (F/B) ratio was markedly higher in the LCP group compared to the HCP group.

#### 3.1.3. Linear Discriminant Analysis Effect Size (LEfSe) Analysis

**Figure 3:**
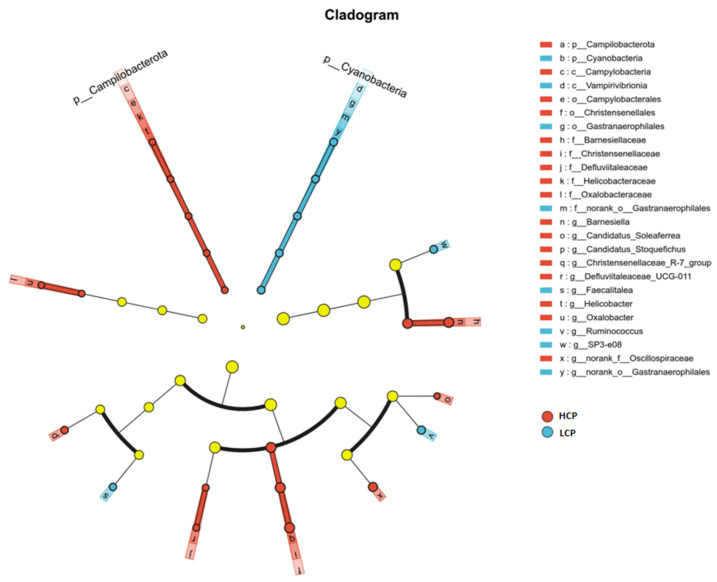
LEfSe analysis of cecal microorganisms. Note: The red branches represent taxa enriched in the HCP group, while the blue branches represent taxa enriched in the LCP group. The circle size corresponds to the relative abundance of each taxon. Different lowercase letters (a, b) indicate significant differences between groups (*p* < 0.05). The yellow circle represents the key bacterial phylum with significant changes.

LEfSe identified significant differences in cecal microbiota composition between the HCP and LCP group (linear discriminant analysis [LDA] score > 2, *p* < 0.05) (Figure 3). Differentially enriched microbiota in the HCP group primarily belonged to the phyla *Bacteroidota*, *Firmicutes*, *Campilobacterota*, and *Proteobacteria*, including the families *Christensenellaceae*, *Barnesiellaceae*, *Helicobacteraceae*, and *Oxalobacteraceae*. Among these, the family *Christensenellaceae* and its subordinate genus Christensenellaceae_R-7_group exhibited the highest LDA scores (3.72 and 3.68, respectively). Enriched taxa in the LCP group mainly derived from Firmicutes and Cyanobacteria, encompassing the family *Erysipelotrichaceae*, the family *Ruminococcaceae*, and the class *Vampirivibrionia*. Specifically, the genus Faecalitalea (*Erysipelotrichaceae*) and the phylum Cyanobacteria had LDA scores of 2.77 and 2.45, respectively. Functionally, the HCP group was characterized by a higher abundance of microbiota associated with short-chain fatty acid (SCFA) biosynthesis and energy metabolism, whereas the LCP group showed enrichment of non-core intestinal microbiota.

### 3.2. Serum Metabolism

#### 3.2.1. Partial Least Squares Discriminant Analysis (PLS-DA)

**Figure 4:**
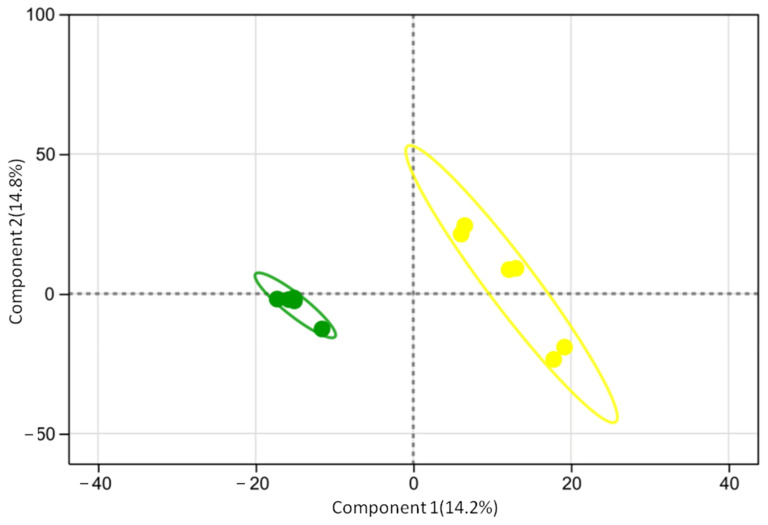
Serum metabolome PLS-DA analysis. Green dots represent the HCP group, and yellow dots represent the LCP group. The ellipses indicate the 95% confidence interval for each group.

The PLS-DA score plot of the serum metabolome revealed a distinct separation trend in the metabolic profiles between the HCP and LCP groups (Figure 4). Samples from the HCP group were distributed in the lower-left area, whereas those from the LCP group were clustered in the upper-right area. The two groups formed separate cluster distributions along component 1 (vertical axis, variance contribution 14.2%) and component 2 (horizontal axis, variance contribution 14.8%), indicating differences in serum metabolites between the two groups.

#### 3.2.2. Comparative Analysis of Serum Metabolites Between Groups

**Figure 5:**
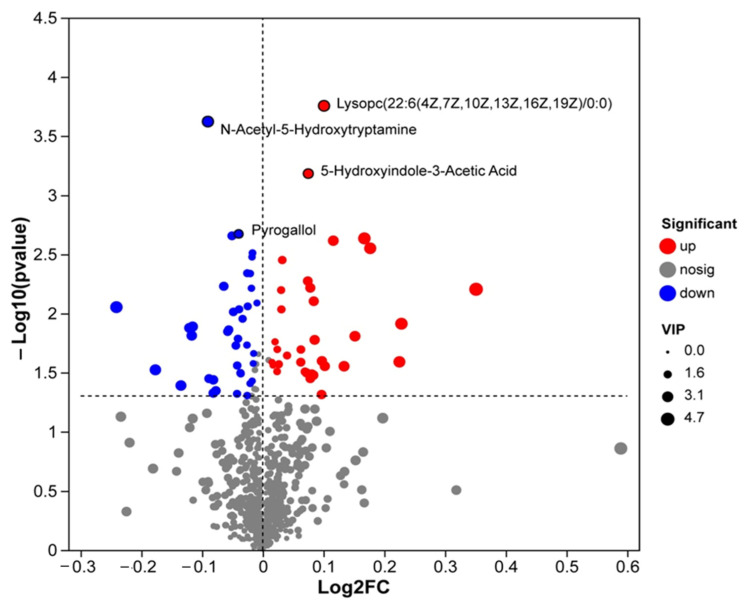
Volcano diagram of differential metabolites in serum metabolome. Note: The names of key differential metabolites are labeled directly on the volcano plot for intuitive reading. The horizontal dashed line indicates the *p* = 0.05 significance threshold, and the vertical dashed line at Log_2_FC = 0 separates upregulated from downregulated metabolites.

The volcano plot of serum metabolome (Figure 5) visualized the differential expressed metabolites (DEMs) between the HCP and LCP groups. Significantly differential metabolites were defined with thresholds of |Log_2_FC| > 1 and −Log10 (*p* value) > 1.3. Among these DEMs, Lysophosphatidylcholine (Lysopc(22:6(4Z,7Z,10Z,13Z,16Z,19Z)/0:0) exhibited the most prominent upregulation (Log_2_FC of ≈0.2, −Log_10_(*p* value) > 3.8). In contrast, N-Acetyl-5-Hydroxytryptamine was downregulated (Log_2_FC ≈ −0.15, −Log_10_(*p* value) = 3.6). Additionally, 5-Hydroxyindole-3-Acetic Acid showed a slight upregulation (Log_2_FC ≈ 0.05, −Log_10_(*p* value) = 3.2), while Pyrogallol was moderately downregulated (Log_2_FC = −0.08, −Log_10_(*p* value) = 2.7).

#### 3.2.3. Variable Importance in Projection (VIP) Value Analysis

**Figure 6:**
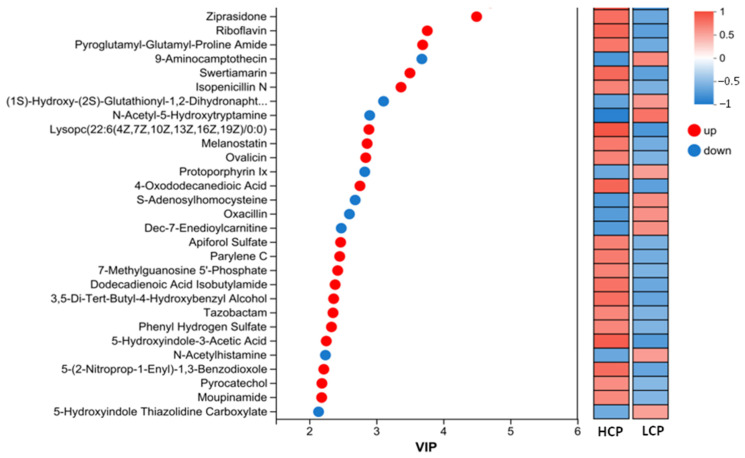
VIP value analysis of differentially abundant metabolites in serum metabolomics.

The VIP plot of serum metabolomics identified a panel of metabolites associated with egg-laying clutch persistence (HCP vs. LCP groups) (Figure 6). Screening criteria prioritized metabolites with high discriminative power (VIP > 2, indicating significant contribution to group separation), clear differential directionality, and functional relevance to egg-laying physiology.

Ranked by VIP values, the top four differentially abundant metabolites were *Ziprasidone* (VIP = 4.496, *p* = 0.0063), *Riboflavin* (VIP = 3.761, *p* = 0.0023), *Pyroglutamyl-Glutamyl-Proline Amide* (VIP = 3.691, *p* = 0.0122), and *9-Aminocamptothecin* (VIP = 3.680, *p* = 0.0089).

In addition to the top-ranked *Ziprasidone* and *Riboflavin*, upregulated metabolites observed in the HCP group included Lysophosphatidylcholine *(Lysopc(22:6(4Z,7Z,10Z,13Z,16Z,19Z)/0:0)*; VIP = 2.890, *p* = 0.00018), *4-Oxododecanedioic Acid* (VIP = 2.756, *p* = 0.0024), *Melanostatin* (VIP = 2.863, *p* = 0.0156), and *Pyrocatechol* (VIP = 2.188, *p* = 0.0486). The identified differential metabolites primarily included Lysophosphatidylcholine (LysoPC), along with various neuroregulatory substances, antioxidants, and lipid signaling molecules. Notably, the majority of these metabolites were significantly upregulated in the HCP group compared to the LCP group (*p* < 0.05).

Downregulated metabolites in the HCP group consisted of *N-Acetyl-5-Hydroxytryptamine* (VIP = 2.901, *p* = 0.00024), *Protoporphyrin IX* (VIP = 2.827, *p* = 0.0408), and *S-Adenosylhomocysteine* (VIP = 2.685, *p* = 0.0129), along with *(1S)-Hydroxy-(2S)-Glutathionyl-1,2-Dihydronaphthalene* (VIP = 3.108, *p* = 0.0300), and *Dec-7-Enedioylcarnitine* (VIP = 2.477, *p* = 0.0133). These metabolites are involved in neurotransmitter precursors, detoxification conjugates, porphyrin synthesis intermediates, and fatty acid transporters, reflecting intergroup differences in neuroendocrine metabolic pathways and specific metabolite biosynthesis.

Notably, *5-Hydroxyindole-3-Acetic Acid* (VIP = 2.254, *p* = 0.00066), a key serotonin metabolite, was significantly upregulated in the HCP group, while N-Acetyl-5-Hydroxytryptamine was downregulated. This directional shift suggests a specific modulation of serotonin metabolism in the HCP group, which may regulate reproductive neuroendocrine function through the gut–ovarian axis.

Furthermore, *7-Methylguanosine* *5′-Phosphate*, a key metabolite involved in RNA capping, was significantly upregulated in the HCP group compared to the LCP group (VIP = 2.424, *p* = 0.0280).

#### 3.2.4. Kyoto Encyclopedia of Genes and Genomes (KEGG) Metabolic Pathway Enrichment Analysis

**Figure 7:**
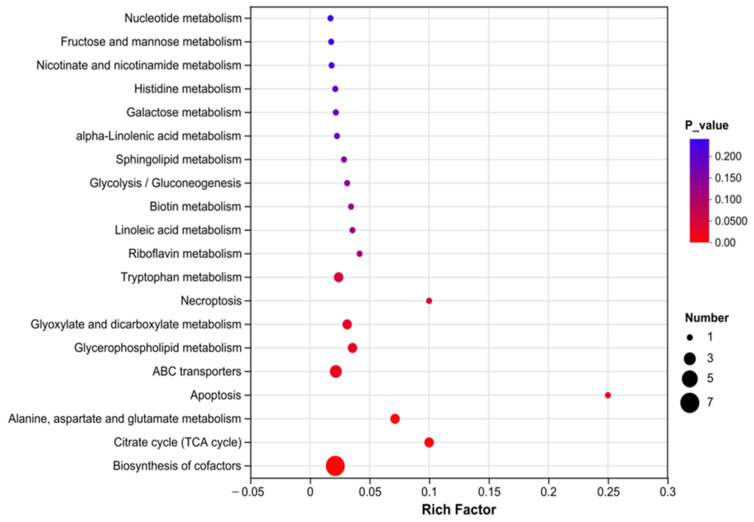
KEGG enrichment analysis of differentially abundant metabolites in serum metabolomics.

KEGG enrichment analysis of the serum metabolome revealed multiple differentially enriched metabolic pathways between the HCP and LCP groups (Figure 7). A total of 20 pathways were significantly enriched (*p* < 0.05). The most significantly enriched pathways, characterized by the smallest *p*-values (approaching 0) and high rich factors, included Biosynthesis of cofactors, Citrate cycle (TCA cycle), Glycerophospholipid metabolism, Glycolysis/Gluconeogenesis, as well as Tryptophan metabolism and Riboflavin metabolism. Furthermore, pathways such as Linoleic acid metabolism and Alanine, aspartate and glutamate metabolism also showed significant enrichment. Concurrently, several other pathways, including Nucleotide metabolism and Fructose and mannose metabolism, were identified but did not reach statistical significance (*p* = 0.200).

### 3.3. Ovarian Transcriptome Results

#### 3.3.1. Ovarian Transcriptome Differential Expression Analysis

**Figure 8:**
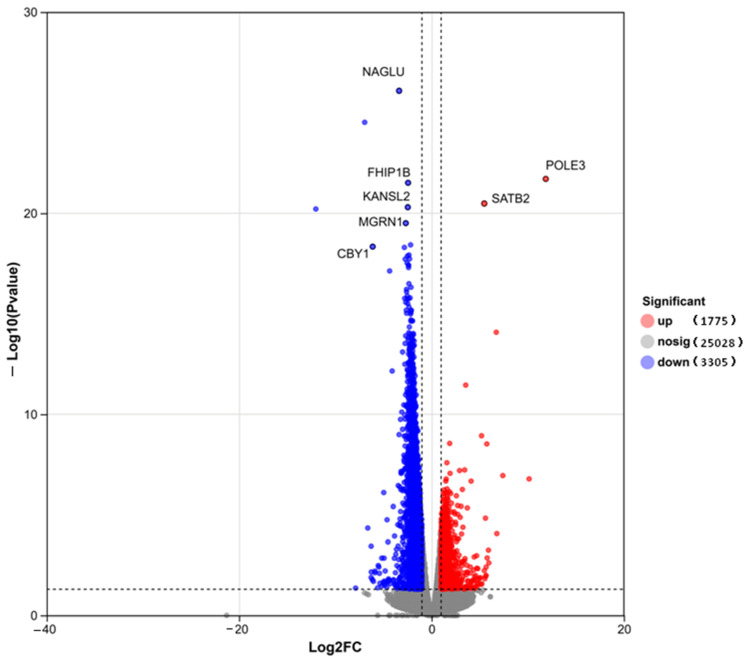
Results of ovarian transcriptome differential expression analysis.

For transcriptome sequencing, three samples were randomly selected from each of the HCP and LCP groups (Figure 8). Differential expression analysis identified a total of 5080 significantly differentially expressed genes (DEGs), comprising 1775 upregulated and 3305 downregulated genes. The volcano plot visualization (Figure 8) illustrates the distribution of all genes, with the x-axis representing theLog_2_FC and the *y*-axis representing the *p*-value. Significantly upregulated genes (*n* = 1775) are marked in red, downregulated genes (*n* = 3305) in blue, and non-significant genes (*n* = 25,028) in gray. Among the most significantly altered genes, *POLE3* and *SATB2* were markedly upregulated, while *NAGLU*, *FHIP1B*, *KANSL2*, *MGRN1*, *LPAR2* and *CBY1* were significantly downregulated.

#### 3.3.2. Gene Ontology (GO) Enrichment Analysis of Differentially Expressed Genes

**Figure 9:**
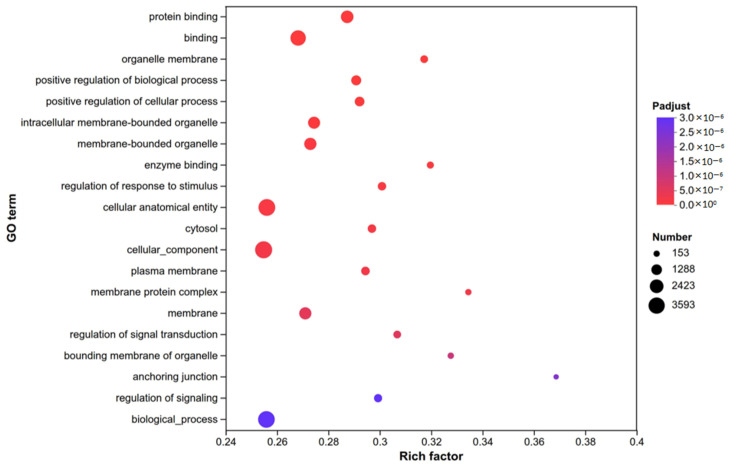
GO enrichment analysis results of transcriptome differentially expressed genes.

GO enrichment analysis identified multiple key functional categories of differentially expressed genes (DEGs) (Figure 9). In terms of molecular functions (MFs), “protein binding” and “enzyme binding” were prominently enriched. For cellular components (CCs), significant categories included “organelle membrane”, “intracellular membrane-bounded organelle”, “membrane-bounded organelle”, “cytosol”, “plasma membrane”, “membrane”, and “organelle bounding membrane”. Regarding biological processes (BPs), enriched terms covered “positive regulation of biological process”, “positive regulation of cellular process”, “regulation of response to stimulus”, “regulation of signal transduction”, and “regulation of signaling”.

#### 3.3.3. KEGG Enrichment Analysis of Ovarian Transcriptome

**Figure 10:**
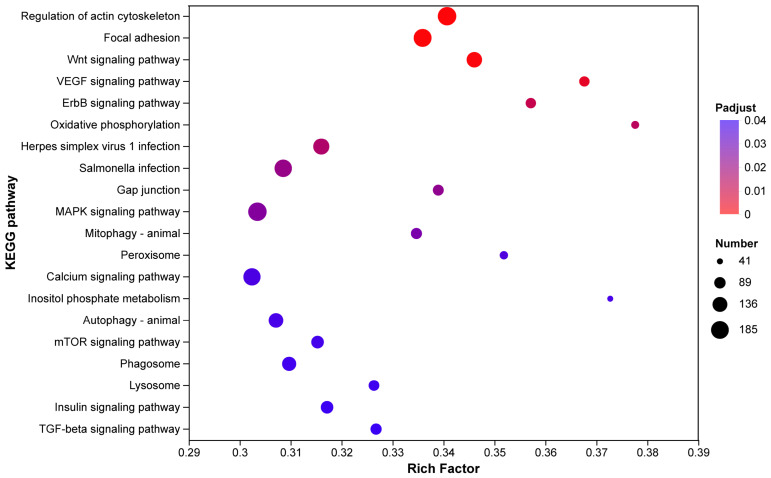
KEGG enrichment analysis results of ovarian transcriptome differentially expressed genes.

KEGG pathway enrichment analysis of the differentially expressed genes (DEGs) revealed significant alterations in several key biological processes (Figure 10). The enriched pathways were primarily categorized into four clusters based on their functional attributes:

Cell Structure and Adhesion: Pathways including “Regulation of actin cytoskeleton” and “Focal adhesion” showed high enrichment significance (Padj < 0.01), with a large number of DEGs mapped to these categories (Figure 10, top red dots).

Signal Transduction: Significant enrichment was observed in multiple signaling pathways, such as “Wnt signaling pathway,” “VEGF signaling pathway,” “ErbB signaling pathway,” “MAPK signaling pathway,” and “Calcium signaling pathway.” Among these, the Wnt and VEGF pathways exhibited the highest Rich Factor values, indicating a strong over-representation of DEGs (Figure 10).

Metabolism: Metabolic pathways, including “Oxidative phosphorylation,” “Arachidonic acid metabolism,” “Glycerolipid metabolism,” and “Glycerophospholipid metabolism,” were significantly enriched. Notably, “Oxidative phosphorylation” contained a substantial number of DEGs (large bubble size), suggesting extensive transcriptional reprogramming in mitochondrial energy metabolism.

Cellular Homeostasis and Response: Pathways related to cellular quality control and growth regulation, such as “Autophagy-animal,” “Lysosome,” “mTOR signaling pathway,” and “Peroxisome,” also displayed significant enrichment (Padj < 0.05).

Overall, the bubble plot illustrates that the HCP and LCP groups differ significantly in genes governing structural integrity, signal transduction, metabolic flux, and cellular homeostasis.

### 3.4. Ovarian Proteomic Analysis

#### 3.4.1. Differential Protein Identification and Characterization

**Figure 11:**
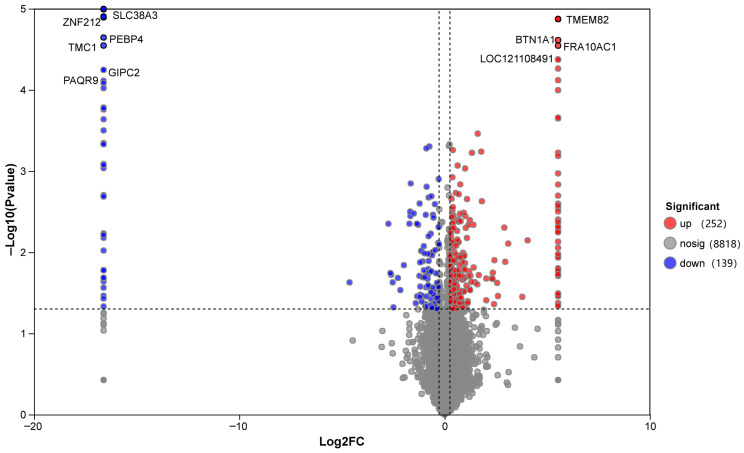
Differential expression analysis results of ovarian proteome. Note: Red dots represent significantly upregulated genes (*n* = 252), blue dots represent significantly downregulated genes (*n* = 139), and gray dots represent non-significantly expressed genes (*n* = 8818). The horizontal dashed line indicates the significance threshold of *p* = 0.05 (corresponding to −log_10_(*p* value) ≈ 1.3). The two vertical dashed lines at Log_2_FC = ±1 indicate the fold change threshold for defining DEGs. The names of key significantly downregulated genes are labeled directly on the plot for intuitive reading.

Differential expression analysis (Figure 11) showed that compared with the LCP group, the HCP group had 252 upregulated proteins and 139 downregulated proteins, totaling 491 differentially expressed proteins (DEPs). The volcano plot (Figure 11) identified core DEPs with the most significant differences: ZNF212, SLC38A3, TMC1, PEBP4, GIPC2, and PAQR9 were downregulated, while BTN1A1, TMEM82, FRA10AC1, and LOC121108491were upregulated. Notably, several of these DEPs are associated with transcriptional regulation, amino acid transport, and lipid metabolism pathways, warranting further investigation into their specific roles in clutch persistence.

#### 3.4.2. GO Enrichment Analysis of Differential Proteins

**Figure 12:**
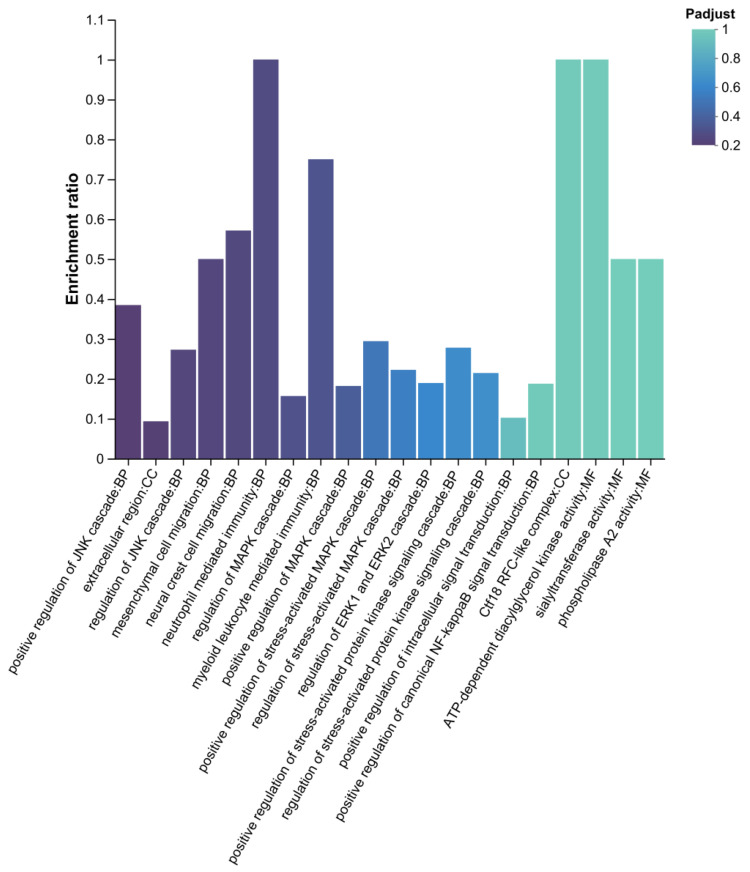
GO enrichment analysis results of differentially expressed proteins in ovarian proteome.

GO enrichment analysis of differential expressed proteins identified functional pathways potentially associated with sustained egg-laying performance (Figure 12). The analysis covered the biological process (BP), cellular component (CC), and molecular function (MF) categories. Key enriched BP terms included “positive regulation of JNK cascade”, regulation of JNK cascade, neutrophil mediated immunity, “regulation of MAPK cascade”, positive regulation of ERK1 and ERK2 cascade, and positive regulation of canonical NF-kappa B signal transduction. Among MF terms, ATP-dependent diacylglycerol kinase activity and phospholipase A2 activity were significantly enriched. The color gradient of the bars corresponds to the adjusted *p*-value (Padjust), with darker shades indicating greater statistical significance.

#### 3.4.3. KEGG Enrichment Analysis of Differential Proteins

**Figure 13:**
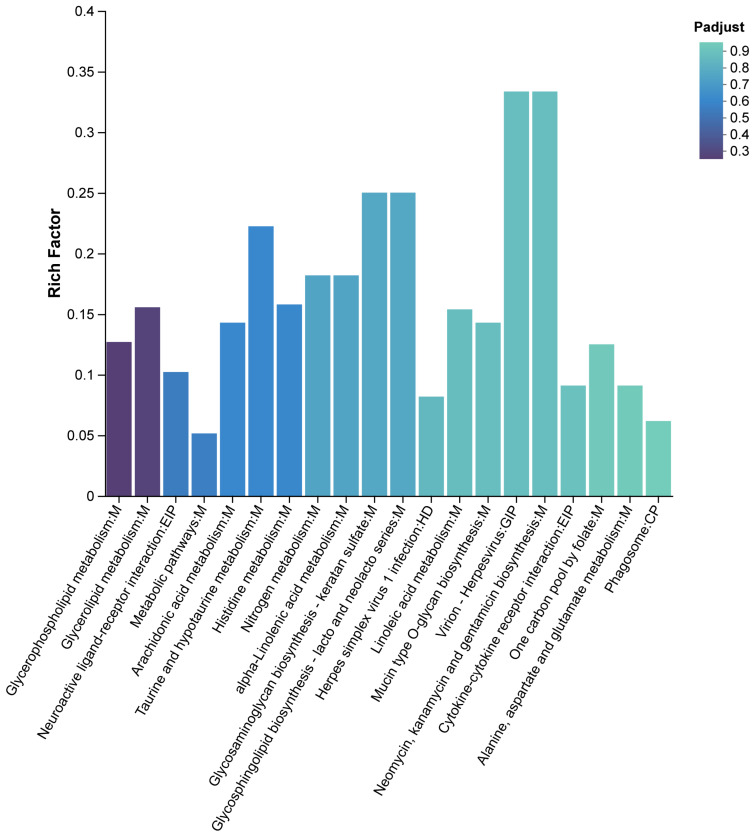
KEGG enrichment analysis results of differentially expressed proteins in ovarian proteome.

Based on KEGG enrichment results of the HCP vs. LCP comparison, and egg-laying clutch-related physiological processes, several functionally relevant pathways were identified (Figure 13). The most significantly pathway was Glycerophospholipid metabolism. Other prominently enriched pathways included Neuroactive ligand–receptor interaction, Arachidonic acid metabolism, and Taurine and hypotaurine metabolism were also significantly enriched. The analysis further revealed enrichment in several other metabolic and signaling pathways, as detailed in Figure 13. The color gradient of the bars corresponds to the adjusted *p*-value (Padjust), with darker shades indicating higher statistical significance. Overall, the enriched pathways were predominantly associated with lipid metabolism, amino acid metabolism, and signal transduction.

### 3.5. Integrated Analysis of Ovarian Transcriptomics and Proteomics

**Figure 14:**
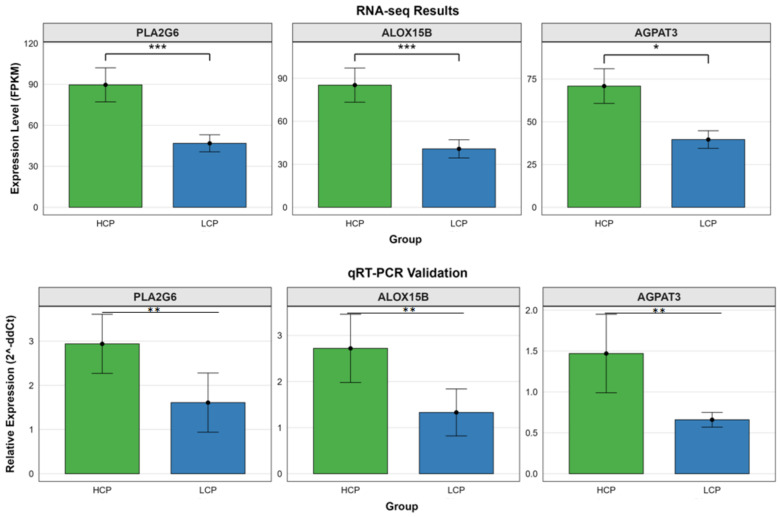

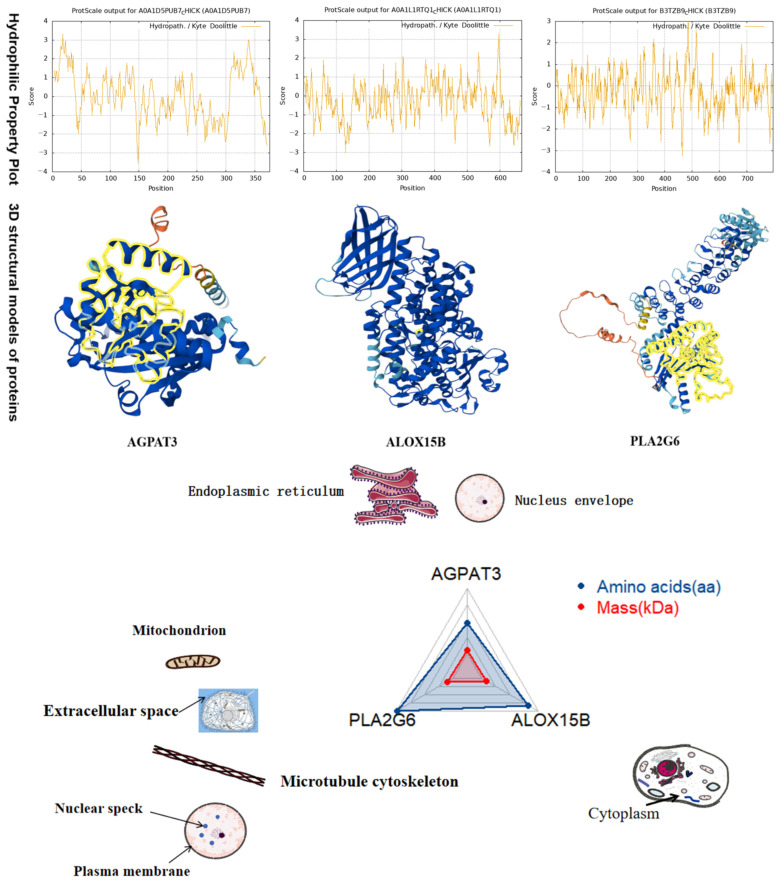
Integrated analysis of ovarian transcriptomic and proteomic common pathways and genes. Note: Red dots represent significantly upregulated genes, blue dots represent significantly downregulated genes, and gray dots represent non-significantly expressed genes. The horizontal dashed line indicates the significance threshold of *p* = 0.05, and the vertical dashed lines at Log_2_FC = ±1 indicate the fold change threshold. The asterisks in the figure denote statistical significance: * *p* < 0.05, ** *p* < 0.01, and *** *p* < 0.001. The names of key genes are labeled directly on the plot for clarity.

Integrated analysis revealed three overlapping pathways between the ovarian proteome and transcriptome: Arachidonic Acid Metabolism, Glycerophospholipid Metabolism and Glycerolipid Metabolism. Additionally, three common DEPs were identified: AGPAT3, ALOX15B and PLA2G6 (Figure 14). Subsequent qRT-PCR validation conducted on ovarian tissues from the HCP and LCP groups confirmed that the mRNA expression levels of *AGPAT3*, *ALOX15B*, and *PLA2G6* were significantly higher in the HCP group, which was consistent with the RNA-Seq data.

For AGPAT3 (1-Acylglycerol-3-Phosphate O-Acyltransferase 3), functional annotation identified multiple conserved domains and conserved peptides. A core domain (aa 90–212) annotated as Phospholipid/glycerol acyltransferase (SMART: SM00563) constitutes the active site for acyltransferase activity, catalyzing acyl group transfer to phospholipid or glycerol substrates to mediate lipid anabolism (functional validation pending in vitro enzyme activity and in vivo metabolomic assays). An Inter-Pro representative domain (aa 62–256, Accession: cd07990) classifies AGPAT3 into the lysophospholipid acyltransferase (LPLATs) family, which participates in glycerophospholipid biosynthesis, exhibiting functional similarity to LCLAT1 to support lipid metabolism regulation. Three unique peptide segments were verified via PeptideAtlas (PAp05852299, PAp05856469, PAp05847477) and aligned to the Saccharomyces cerevisiae proteome (UP000000539): PROTEOMICS 162-179 (sequence: LADYPEYMWFLLYCEGTR), PROTEOMICS 187-195 (sequence: ISMEVAESK), and PROTEOMICS 282-303 (sequence: DALQEMYNOQEGTFPGQQFKPPR). These peptides serve as specific biomarkers for targeted proteomic detection and quantification. For ALOX15B (Arachidonate 15-Lipoxygenase Type B), multiple functional sites critical for biological activity were identified, including two Ca^2+^ binding sites (aa 17 and 76) and four Fe^2+^/Fe^3+^ binding sites (aa 364, 369, 544 and 677). The metal-ion binding sites may mediate iron transport, storage, or catalytic reactions. Notably, the amino acid at position 99 is essential for stabilizing ALOX15B interaction with cortical actin-like protein 1 (COTL1)—a regulator of cytoskeletal dynamics and signal transduction—suggesting a key role in modulating ALOX15B-mediated cellular pathways.

PLA2G6 (Phospholipase A2 Group VI) contains six domains, including one PNPLA domain (aa 472–656) and five ANK (ankyrin) repeat domains. Three conserved motifs and two catalytic sites were annotated via PROSITE (PRU01161): MOTIF 476-481 (pattern: GXGXXG, potentially energy metabolism-related), MOTIF 508-512 (pattern: GXSG, mediating ligand interaction and conformational stability), MOTIF 643-645 (pattern: DGA/G, linked to catalysis), ACT_SITE 510 (nucleophile), and ACT_SITE 643 (proton acceptor). Functional characterization requires experimental validation, such as site-directed mutagenesis and enzyme activity assays.

Key physicochemical properties of the three proteins are summarized as follows: AGPAT3 (376 aa, 43.6 kDa, pI 8.76) localizes primarily to the endoplasmic reticulum; ALOX15B (667 aa, 75.9 kDa, pI 6.53) is cytoplasmic; PLA2G6 (796 aa, 88.4 kDa, pI 7.07) distributes across multiple compartments (extracellular space, microtubule cytoskeleton, mitochondrion, nuclear speck, and plasma membrane). Notably, protein hydrophilicity data were incomplete in the original dataset.

### 3.6. Ovarian Metabolomic Analysis

#### 3.6.1. Data Preprocessing and Quality Control

Table 3 summarizes the preprocessing results of ovarian metabolomics data across different ion modes. In positive ion mode (Pos), the number of effective peaks decreased from 4454 to 3131 post-preprocessing, while the metabolite identification rate increased from 12.17% to 15.3%. In negative ion mode (Neg), effective peaks reduced from 4296 to 3336, with the identification rate rising from 6.33% to 7.37%. For the mixed mode, total effective peaks declined from 8750 to 6467, and the identification rate improved from 9.3% to 11.21%. Quality control (QC) processing significantly enhanced metabolite identification efficiency across all three modes, with an average increase of over 2 percentage points. This confirms that the preprocessing workflow effectively filtered noise peaks while preserving target metabolite information. Subsequent analyses were performed using the preprocessed data matrix (see “origin” in Table 3).

#### 3.6.2. Expression Level Data Processing

Table 4 summarizes the ovarian metabolomic data processing results. In positive ion mode, 3131 mass spectrometry peaks were detected, with 479 metabolites identified, including 381 annotated in public databases and 202 matched to the KEGG database. In negative ion mode, 3336 peaks were captured, corresponding to 246 identified metabolites (211 in public databases and 126 with KEGG annotations). Mixed mode integration yielded 6467 total peaks and 725 identified metabolites, of which 592 were annotated in public databases and 328 in KEGG. Unidentified peaks accounted for over 80% of total peaks across all modes (Pos: 2652, Neg: 3090, mix: 5742), reflecting the abundance of uncharacterized metabolite information in non-targeted metabolomics. These results provide a robust data foundation for subsequent differential metabolites screening. Notably, the mixed mode yielded 328 KEGG-annotated metabolites, accounting for 45.2% of all identified metabolites, facilitating downstream pathway enrichment analyses.

#### 3.6.3. Partial Least Squares-Discriminant Analysis (PLS-DA)

**Figure 15:**
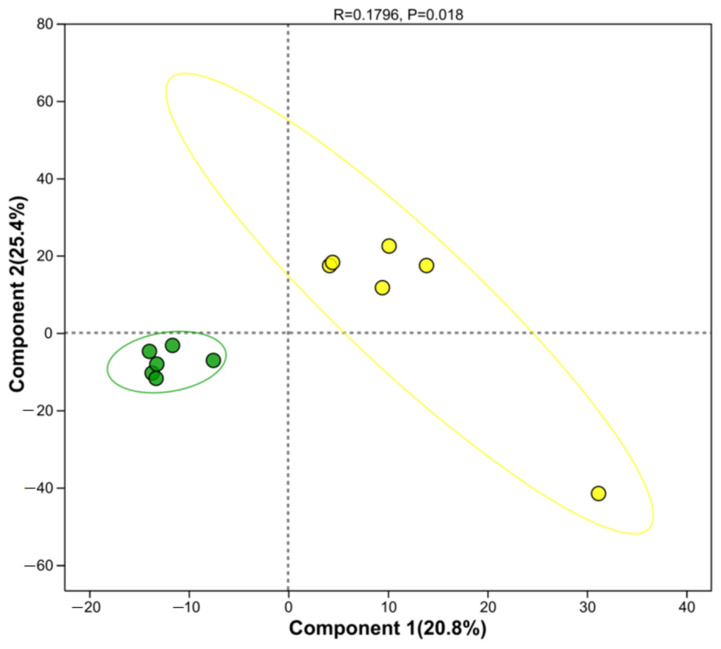
PLS-DA score plot of ovarian metabolomic profiles. Note: Green dots represent the HCP group, and yellow dots represent the LCP group. The ellipses indicate the 95% confidence interval for each group.

The robust separation observed in the PLS-DA model (Figure 15), driven primarily by Component 1, likely reflects fundamental metabolic divergences essential for sustained egg production. Central to this distinction is lipid homeostasis. Beyond metabolic substrate availability, the variance captured by Component 2 points to differences in regulatory signaling. This axis appears to mirror neuroendocrine modulation, consistent with the enrichment of neuroactive ligand–receptor interaction pathways, as well as prostaglandin-mediated ovulation rhythms potentially governed by key enzymes such as ALOX15B and PLA2G6. Collectively, these findings illustrate that ovarian metabolism is tightly shaped by egg-laying performance.

#### 3.6.4. Analysis of Differential Metabolites

**Figure 16:**
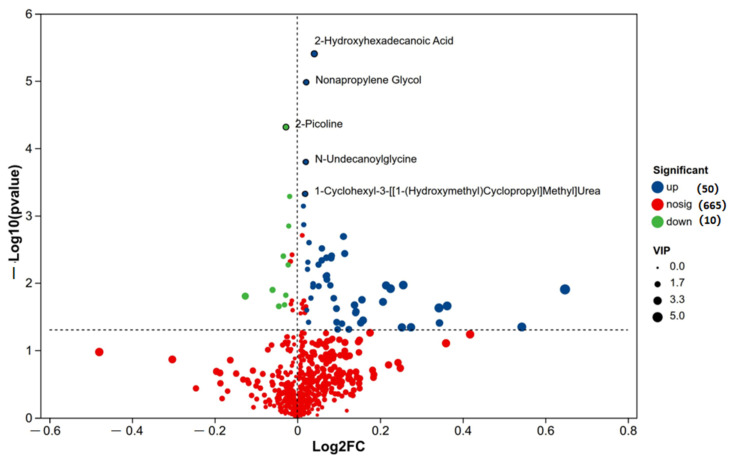
Volcano plot of ovarian differential metabolites. Note: The horizontal axis represents the logarithmic fold change in metabolite expression between the two groups; the vertical axis represents the statistical significance of expression changes (−log_10_(*p*-value)), with higher values indicating more significant differences. Both axes use logarithmic transformation. Each dot denotes a single metabolite, with dot size reflecting the VIP value. By default, red dots represent significantly upregulated metabolites, blue dots represent significantly downregulated metabolites and black dots represent metabolites with non-significant differences. The horizontal dashed line represents the significance threshold of *p* = 0.05 (corresponding to −log_10_(*p*) = 1), above which metabolites show statistically significant differences. The vertical dashed line at log_2_FC = 0 is the baseline indicating no fold change, separating upregulated (right) and downregulated (left) metabolites.

The ovarian metabolome volcano plot (Figure 16) clearly distinguished metabolic profiles between the HCP and LCP groups. A total of 60 metabolites were significantly differentially expressed (DEMs): 50 were upregulated, and 10 were downregulated in the HCP group relative to the LCP group.

Among the most significantly upregulated DEMs (red dots), 2-hydroxyhexadecanoic acid, nonylpropanediol, N-undecanoylglycine, and 1-cyclohexyl-3-[[(1-hydroxymethyl) cyclopropyl] methyl] urea were identified. In contrast, 2-picoline (2-methylpyridine) was the most prominently downregulated DEM (blue dot). Metabolites represented by black dots showed no significant intergroup differences. These specific DEMs, particularly those involved in lipid and amino acid metabolism, were prioritized for further correlation analysis with microbial and genomic data.

#### 3.6.5. VIP Value-Based Metabolite Screening and Functional

**Figure 17:**
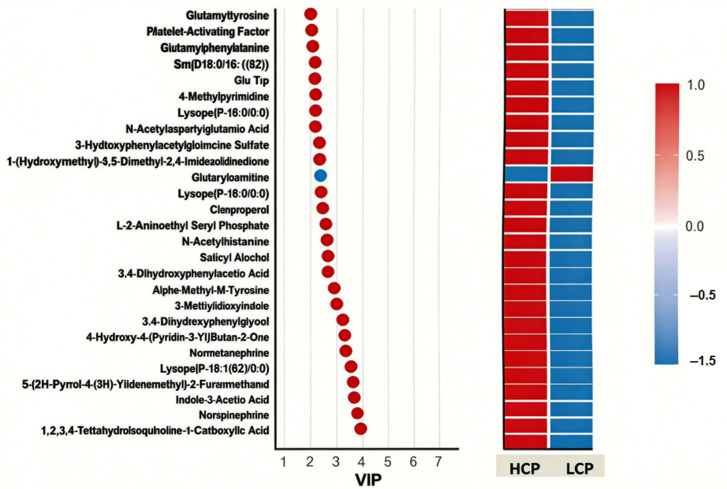
VIP score plot of ovarian metabolites for egg-laying clutch trait discrimination.

To identify key metabolites mediating egg-laying clutch persistence, we screened candidates with high discriminatory power using a VIP score plot (Figure 17), where dot color indicates VIP value magnitude (red = high VIP, blue = low VIP). The screening was further refined by integrating pathway biological functions and intergroup expression differences. The analysis identified several top-ranking metabolites contributing to the separation between groups. Upregulated metabolites in the HCP group (red dots) with the highest VIP scores included Norepinephrine, Indole-3-Acetic Acid (IAA), Platelet-Activating Factor (PAF), and specific lysophospholipids such as LysoPC (P-18:1(9Z)/0:0), LysoPE (P-16:0/0:0), and LysoPE (P-18:0/0:0). Conversely, 1-(hydroxymethyl)-5,5-dimethyl-2,4-imidazolidinedione was identified as a key downregulated metabolite (blue dot) in the HCP group. These high-VIP metabolites, spanning categories of lipid signaling, neuroregulation, and iron metabolism, were selected as core candidates for subsequent multi-omics correlation analysis.

#### 3.6.6. KEGG Pathway Enrichment Analysis of Differential Metabolites

**Figure 18:**
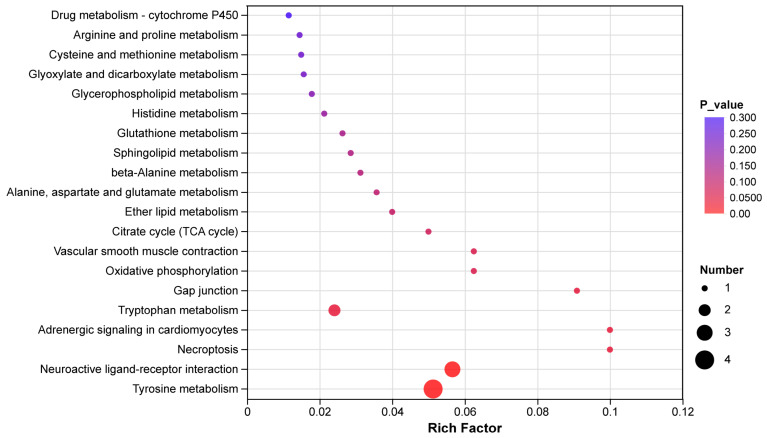
KEGG enrichment plot of ovarian metabolome. The vertical axis denotes KEGG pathways. Bubble size indicates the number of DEMs enriched in the pathway; bubble color represents the statistical significance of enrichment (darker color corresponds to a smaller *p*-value).

To dissect the metabolic regulatory networks underlying egg-laying clutch persistence, KEGG pathway enrichment analysis was performed on ovarian differentially expressed metabolites (DEMs) between the HCP and LCP groups (Figure 18). The analysis revealed that Glycerophospholipid metabolism was the most significantly enriched pathway, characterized by a low *p*-value and high Rich Factor (large bubble size in Figure 18), indicating its central role in the observed metabolic divergence. Other key pathways significantly enriched included Arginine and proline metabolism, Citrate cycle (TCA cycle), and Tyrosine metabolism. Additionally, pathways related to signal transduction, such as Neuroactive ligand–receptor interaction and Adrenergic signaling in cardiomyocytes, were also identified. These enriched pathways highlight the involvement of lipid metabolism, energy production, and neuroendocrine regulation in distinguishing the two phenotypes.

### 3.7. Serum-Targeted Metabolomics Validation

To validate the multi-omics-derived hypothesis that “the gut–ovarian axis regulates egg-laying clutch persistence in laying hens via lipid mediators”, targeted metabolomics was performed on serum samples from HCP and LCP groups. This analysis systematically characterized intergroup differences in serum metabolic phenotypes, core lipid mediator abundance, and associated regulatory pathways, with key findings presented below.

#### 3.7.1. Data Quality Control and Preprocessing

Raw quantitative data for 956 metabolites were acquired via targeted detection. Post-quality control (QC) and preprocessing—including missing values imputation (half the minimum detected value) and outlier filtering (coefficient of variation [CV] > 30% excluded)—of all 956 metabolites retained eligibility for downstream statistical analysis resulted in no significant data loss or technical artifacts. Intra-group metabolite quantification exhibited a relative standard deviation (RSD) < 20%, confirming excellent detection reproducibility and suitability for intergroup comparison.

#### 3.7.2. Intergroup Segregation of Serum Metabolic Phenotypes

##### Orthogonal Partial Least Squares-Discriminant Analysis (OPLS-DA)

**Figure 19:**
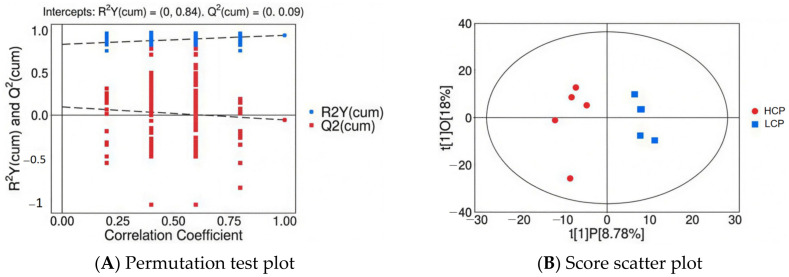
OPLS-DA model for HCP vs. LCP serum metabolic profiles: (**A**) Permutation test plot: The dashed line represents the regression line of Q^2^ values. (**B**) Score scatter plot: The ellipse represents the 95% confidence interval.

A supervised OPLS-DA model was constructed to prioritize intergroup metabolic differences (Figure 19). Model parameters were: principal components configuration 1 + 1 + 0, cumulative explained variance of X-variables (R^2^X(cum)) = 0.268, cumulative explained variance of grouping variables (R^2^Y(cum)) = 0.944, and cumulative predictive ability (Q^2^(cum)) = −0.057. The high R^2^Y(cum) (94.4%) indicated a strong correlation between serum metabolic profiles and egg-laying persistence phenotypes. Although Q^2^(cum) was negative—attributed to the small sample size (*n* = 5/group)—200-permutation test validation (Figure 19A) ruled out over-fitting: R^2^Y and Q^2^ values of permuted models were consistently lower than the original model, with *p* value for R^2^Y > 0.05 (126/200 permutations), and Q^2^ > 0.05 (23/200 permutations l). The OPLS-DA score plot (Figure 19B) demonstrated complete spatial segregation of the two groups along the predictive component t[1]P axis, with tight intra-group clustering along the orthogonal component t[1]O axis. This confirms phenotype-specific serum metabolic signatures and robust intra-group consistency—supporting the role of systemic lipid metabolism in mediating egg-laying persistence.

#### 3.7.3. Core Differential Lipid Mediators

**Figure 20:**
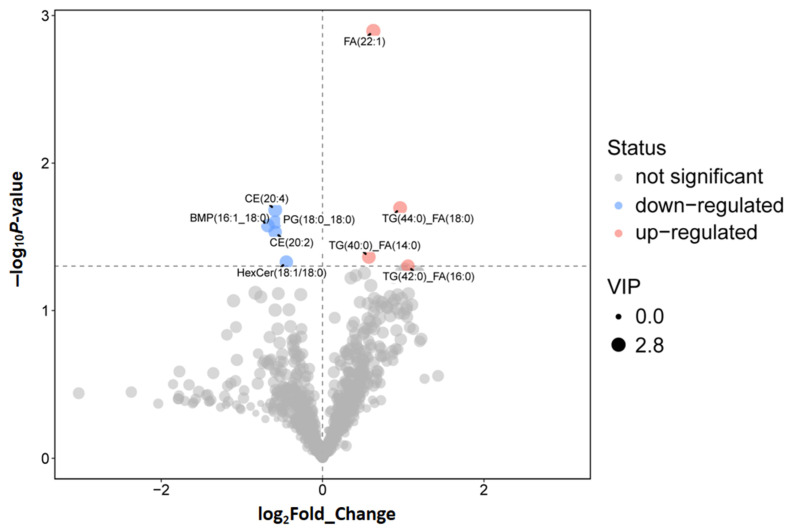
Volcano plot of serum differential lipid mediators.

Screening and Functional Traits Combined univariate (Student’s t-test) and multivariate (OPLS-DA VIP value) analyses identified nine significant differential metabolites (DEMs) using the criteria: VIP > 1, *p* < 0.05, and |Log_2_FC| > 0.58 (Figure 20). All nine DEMs were lipids or lipid derivatives, with four upregulated and five downregulated in the HCP group relative to LCP.

HCP-upregulated lipid mediators: Long-chain fatty acid FA(22:1) (VIP = 2.798, *p* = 0.001, FC = 1.549, Log_2_FC = 0.631), triglyceride(TGs) including TG (40:0)_FA(14:0) (VIP = 2.452, *p* = 0.043, FC = 1.49, Log_2_FC = 0.575), TG(42:0)_FA(16:0), and TG(44:0)_FA(18:0). These TGs and FA (22:1) serve as high-energy substrates; their elevated levels in HCP provide a continuous energy supply for follicular development and ovulation—addressing the high ATP demand of sustained egg-laying observed in the ovarian transcriptome’s oxidative phosphorylation pathway.

LCP-upregulated lipid mediators: Cholesterol esters (CEs) CE(20:4) (VIP = 2.624, *p* = 0.021, FC = 0.666, Log_2_FC = −0.587) and CE(20:2) (VIP = 2.337, *p* = 0.029, FC = 0.666, Log_2_FC = −0.586), phosphatidylglycerol PG(18:0_18:0) (VIP = 2.554, *p* = 0.025, FC = 0.667, Log_2_FC = −0.585), diacylglycerophosphate BMP(16:1_18:0) (VIP = 2.47, *p* = 0.027, FC = 0.625, Log_2_FC = −0.677), and ceramide HexCer(18:1/18:0) (VIP = 2.347, *p* = 0.047, FC = 0.733, Log_2_FC = −0.448). Critically, CE(20:4)—the cholesterol ester form of arachidonic acid (ARA)—is a key precursor for ovarian prostaglandin synthesis. Its higher abundance in HCP aligns with upregulated PLA2G6 (phospholipase A2) in our transcriptome–proteome integration, ensuring sufficient ARA release for prostaglandin-mediated ovulation rhythm regulation.

#### 3.7.4. Functional Association and Pathway Enrichment of Differential Lipids

##### Hierarchical Clustering Lipids’ Expression Patterns

**Figure 21:**
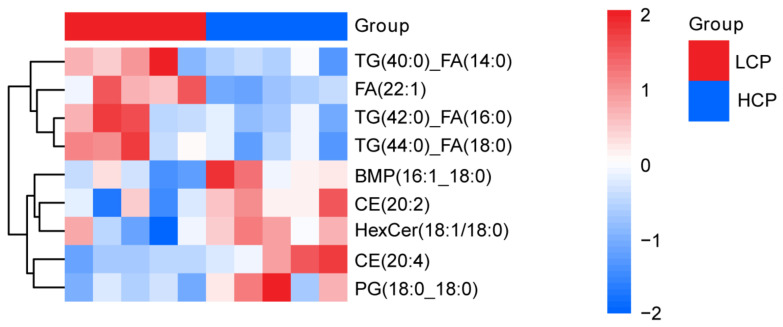
Hierarchical clustering heatmap of serum differential lipids.

Hierarchical clustering analysis (Euclidean distance, complete linkage) of the nine differential lipids yielded two distinct clusters perfectly matching the grouping (Figure 21). At the metabolite level, FA(22:1) and TG subtypes (TG(40:0)_FA(14:0), TG(42:0)_FA(16:0)) formed a LCP-dominant branch, reflecting dysregulated energy storage; CE subtypes (CE(20:4), CE(20:2)), PG(18:0_18:0), and HexCer (18:1/18:0) formed a HCP-dominant branch, indicative of enhanced lipid precursor reserve. This pattern supports a phenotype-linked metabolic divergence: HCP serum lipid metabolism prioritizes energy substrate supply (for high-frequency follicular development) and lipid signaling precursor storage (for ovulation regulation), whereas LCP exhibits disrupted lipid homeostasis—consistent with the ovarian metabolome’s observation of inefficient energy utilization in LCP.

##### KEGG Pathway Enrichment

Mapping these different lipids to the chicken (*Gallus gallus*) KEGG database identified two significantly enriched core pathways: gga00561 Glycerolipid Metabolism and gga00100 Steroid Biosynthesis—both overlapping with the ovarian transcriptome–proteome integrated pathways. Glycerolipid Metabolism (gga00561): Glycerol-3-phosphate (KEGG: C00422) showed elevated abundance in HCP. This aligns with upregulated AGPAT3 (glycerophospholipid synthase) in our multi-omics integration, indicating active phospholipid biosynthesis in HCP serum, providing structural precursors for follicular membrane formation and lipid signaling molecule synthesis. Steroid Biosynthesis (gga00100): Differential expression of cholesterol metabolism intermediate (KEGG: C02530) in this pathway correlates with CE (20:4) and CE (20:2) abundance.

## 4. Discussion

### 4.1. Intestinal Microbiota: The Initiation Hub of Regulatory Signals

Traditional “ovarian-centric theory” emphasizes follicular development, hormone secretion, and ovarian function as core determinants of egg-laying performance [23,24,25]. However, this framework fails to explain the observed correlation between intestinal health and egg-laying traits, such as intergroup differences in ovarian morphology and reproductive performance in laying hens. A critical limitation lies in its neglect of regulatory inputs from peripheral systems, particularly the intestine. To address this gap, we propose an integrated “gut–ovarian axis” model mediated by a lipid mediator network in laying hens, whose core logic revolves around the three-stage cascade: intestinal microbiota–serum metabolites–ovarian function.

The intestinal microbiota exhibited distinct compositional and functional divergence between the HCP and LCP groups, forming the initial regulatory layer. In HCP, functionally significant γ-Proteobacteria (including Hydrogenophaga and Methylobacteriaceae) were enriched, which enhance energy metabolism, stabilize membrane structure, and reduce the production of deleterious metabolites—collectively creating a favorable microenvironment for supported lipid-mediated signal transmission. In contrast, LCP is dominated by presumably environmental taxa, which correlate with disrupted intestinal homeostasis, impaired intestinal barrier function, and increased risk of ovarian damage. These findings support the intestinal microbiota as a “metabolic signal generator” that modulates extraintestinal tissues via metabolite secretion [26]. Specifically, it produces or modifies lipid precursors that serve as initial signaling molecules for downstream regulatory processes. Our serum target metabolome data provide critical validation for this cascade: HCP exhibits significantly higher serum concentrations of cholesterol ester CE (20:4) compared to LCP. As the cholesterol ester form of arachidonic acid (ARA), CE (20:4) is a core carrier that stably circulates in serum after ARA synthesis by the intestinal microbiota. Combined with the functional trait of HCP-enriched γ-proteobacteria, this confirms a clear regulatory chain: γ-proteobacteria generate ARA via metabolic activities, which is then incorporated into serum circulation as CE (20:4). This provides direct molecular carrier evidence for gut–ovary cross-organ signal transmission, addressing the limitation of earlier omics studies that could only infer associations without empirical support for the serum intermediate link. Furthermore, recent evidence suggests that such gut-derived lipid precursors are essential for the metabolic reprogramming required during follicle selection, acting as the material basis for sustained reproduction [27,28].

### 4.2. Serum Metabolites: The Cross-Organ Signaling Hub

Lysophosphatidylcholine (22:6) in HCP serum exhibits a high VIP value of 4.5. It maintains the continuity and coordination of follicular development, as well as cellular functional stability, by activating the ovarian G protein-coupled receptor LPAR2 via the LPA-LPAR2 signaling axis—ultimately supporting continuous ovulation and sequential egg-laying in hens. LysoPC is a bioactive lipid mediator produced by phospholipase A2-catalyzed hydrolysis of phosphatidylcholine (PC), which is abundant in the circulatory system; its fluctuations dynamically reflect cholesterol concentrations [29]. Specifically, LysoPC (22:6) is a Lysophosphatidylcholine subtype carrying docosahexaenoic acid (DHA), which releases DHA under the action of phospholipase (PLA1/PLA2). DHA not only reduces triglyceride concentrations and cardiovascular disease risk [30] but also mediates vasodilation to regulate blood pressure [31]—processes that indirectly support ovarian blood supply and nutrient delivery.

LPAR2 is a G protein-coupled receptor, participating in cell development, proliferation, and apoptosis, with critical roles in follicular development. A SNP (rs410670692) in the 3′UTR of LPAR2 correlates with litter size in small-tailed Han sheep: individuals with the TT genotype exhibit higher LPAR2 expression. This SNP acts as a targeted regulatory element of miR-939-5p, which downregulates LPAR2 mRNA and protein levels by interfering with miR-939-5p binding. In sheep granulosa cells, overexpression of miR-939-5p inhibits granulosa proliferation and promotes apoptosis by blocking the LPAR2-dependent PI3K/Akt signaling pathway, thereby indirectly affecting litter size [32]. Beyond ruminants, LPAR2 has been implicated in pan-cancer prognosis, Muscovy duck egg-laying regulation [33], and broiler testis transcriptional response to heat stress [34]. In ovarian physiology, LPAR2 expression in follicular theca cells may mediate lysophosphatidic acid (LPA)-dependent follicular regulation, including participation in cell proliferation, differentiation, and steroid synthesis [35]. Its high mRNA expression in atretic follicles suggests a potential role in follicular atresia. Additionally, theca cells transduce LPA signals via LPAR2 and may cooperate with granulosa cells to regulate intrafollicular hormonal balance, thereby influencing follicular development and dominant follicle selection. This mechanism confirms that LysoPC (22:6) acts as both a downstream metabolite of Bacteroidetes metabolism and an upstream activator of ovarian lipid signaling, forming a complete “microbiota metabolism–serum transmission–ovarian response” signaling chain in HCP. Five other serum lipid metabolites—BMP (16:1_18:0), CE (20:4), CE (20:2), HexCer (18:1/18:0), and PG (18:0_18:0)—synergize with LysoPC (22:6) to constitute the core intermediate carrier network for gut–ovary cross-organ regulation, forming a coordinated functional cascade. Our serum-targeted metabolomics data revealed that the HCP phenotype is associated with a specific profile of differential lipids. Rather than functioning in isolation, these lipids constitute a functionally coordinated network that collectively establishes a metabolic microenvironment conducive to sustained follicular development [36,37]. The operation of this network follows an axis logic of “intestinal synthesis, serum transport, and ovarian response.”

In HCP hens, an enriched intestinal functional microbiota provides biosynthetic precursors for these lipids. For instance, γ-Proteobacteria may conjugate dietary fatty acids with cholesterol to generate cholesteryl esters CE (20:4) and CE (20:2), while Bacteroidota participate in phospholipid metabolism to produce BMP and phosphatidylglycerol PG (18:0_18:0) and regulate sphingolipid synthesis to yield hexosylceramide HexCer (18:1/18:0). These synthetic processes are closely linked to the maintenance of intestinal homeostasis and enhancement of barrier function, laying the material foundation for inter-organ signaling. Upon entering the circulation, these lipids achieve stable transport via their distinct structural properties: CEs form complexes with lipoproteins, BMP maintains its biological activity, and PG and HexCer integrate into the serum lipid network, collectively forming a cooperatively transported lipid signaling system.

Among the various lipids, LysoPC (22:6) acts as a central hub due to its highest VIP score. Existing literature indicates that LysoPC can serve as a precursor for the bioactive lipid LPA [38]. The elevated level of LysoPC (22:6) observed in the HCP group may activate local ovarian LPA signaling pathways, thereby providing crucial mitogenic and survival signals to follicular cells to support their homeostasis.

Other lipids undergo functional transformation and act synergistically upon reaching the ovary, providing multidimensional support for folliculogenesis. CE (20:4) and CE (20:2) serve a dual physiological purpose: they act as cholesterol reservoirs essential for steroid hormone synthesis [39], and the arachidonic acid (20:4) released upon their hydrolysis is a direct precursor for eicosanoid signaling molecules like prostaglandins, which are key local regulators of processes such as ovulation. BMP (16:1_18:0) is a specific phospholipid predominantly localized to endosomal/lysosomal membranes [36,37,40] and plays a central role in maintaining lysosomal function, membrane trafficking, and sphingolipid degradation [37,40]. Its enrichment in the HCP group may reflect more active membrane remodeling and cargo transport in ovarian cells, supporting the high turnover of follicular growth and atresia. Studies also indicate that BMP species containing specific polyunsaturated fatty acids possess antioxidant properties that protect lysosomal membranes [38], which may help maintain the health of oocytes and granulosa cells in a metabolically active environment. As a key molecule in sphingolipid metabolism, changes in HexCer may indirectly regulate follicular growth and atresia dynamics by influencing the physical properties of ovarian cell membranes and downstream signal transduction. PG (18:0_18:0) is a crucial precursor for cardiolipin in the mitochondrial inner membrane, which is essential for maintaining mitochondrial structure and oxidative phosphorylation function [38]. Differences in its level may indicate a more efficient or stable energy metabolism state in the ovarian cells of the HCP group, providing ample ATP support for the biosynthesis and homeostatic maintenance required for sustained egg production.

In summary, the serum lipid profile associated with the HCP phenotype represents a physiologically coherent “metabolic support program.” This program is orchestrated with LysoPC (22:6) as the key systemic signaling mediator, CEs supplying hormone synthesis substrates and eicosanoid precursors, BMP supporting intracellular membrane trafficking and homeostasis, HexCer involved in membrane structure and signal fine-tuning, and PG linked to foundational energy metabolism. These lipids are coordinately delivered to the ovary via the bloodstream, forming a multidimensional, synergistic metabolic support network. The stable operation of this network is likely a key systemic metabolic basis enabling HCP hens to maintain efficient waves of follicular development and achieve high clutch persistence. This finding also provides a new line of evidence at the metabolome level for the functional integration of the “gut–serum–ovary” axis. Future studies are needed to functionally validate the causal roles of specific lipids within this axis and in clutch persistence by experimentally modulating their levels.

Importantly, beyond simple substrate supply, our data suggest a vascular–metabolic coupling mechanism. The enrichment of arginine-related metabolites and specific glycerophospholipids in HCP supports the hypothesis that estrogen’s rapid non-genomic effects on ovarian vasodilation depend on an ERα-Arginine-eNOS-NO axis [41,42]. We propose that the specific lipid profile in HCP ensures robust ovarian perfusion and nutrient delivery essential for high-frequency ovulation, whereas the depletion of these lipids in LCP may lead to vascular insufficiency, starving the follicles despite adequate hormonal stimulation.

The KEGG pathway annotation of serum-targeted metabolomics confirms that these differential lipids are significantly enriched in the Glycolipid metabolism pathway (gga00561). Glycerol-3-phosphate (KEGG: C00422), a core intermediate in phospholipid synthesis, exhibits high abundance in HCP serum—consistent with the ovarian omics finding that the PLA2G6-ALOX15B axis regulates phospholipid metabolism. The differential distribution of serum lipid intermediate products provide direct substrate source of the ovarian PLA2G6-ALOX15B axis: PLA2G6 catalyzes the release of serum-derived phospholipids into ovarian cells, which are then metabolized by ALOX15B to regulate follicular development; AGPAT3 utilizes serum lipid precursors for phospholipid remodeling, stabilizing the follicular membrane structure.

Collectively, our findings indicate that the high clutch persistence observed in the DLCD Group is associated with a coordinated gut–ovary regulatory axis. Within this framework, specific serum lipids of gut microbiota—originating with LysoPC (22:6) as the signaling molecule, supported by BMP, CE (20:4), CE (20:2), HexCer (18:1/18:0), and PG (18:0_18:0) as key intermediate carriers—are precisely transported to the ovary. At this site, they activate critical receptors, functionally synergize with upregulated genes, and thereby provide integrated multi-dimensional support encompassing energy supply, signal transmission, and structural integrity. This coordinated sequence from “intestinal synthesis through serum transport to ovarian response” ensures the continuity of follicular development and ovulation, constituting a key mechanistic basis for the high clutch persistence phenotype.

### 4.3. Ovarian Lipid Metabolic Pathways: The Functional Core

Cross-omics data analysis identifies the PLA2G6-ALOX15B-AGPAT3 pathway as a key regulatory hub for follicular development, achieving synergy via metabolic flux guidance and functional effect coupling. As the pathway’s initiation node, *PLA2G6* is significantly upregulated by 1.8-fold. It encodes calcium-independent phospholipase A2β (iPLA2β), which drives the intracellular release of ARA from membrane phospholipids—providing core substrates for downstream prostaglandin synthesis that promotes follicular maturation, thereby defining the direction of metabolic flux. iPLA2β also releases DHA (22:6n-3), which participates in inflammation, immunity and vasodilation [43,44]. ARA, as a key precursor, is converted into inflammatory mediators via cyclooxygenase or lipoxygenase [45]—mediating ovulation and follicular survival.

*ALOX15B* (a lipoxygenase family member) catalyzes the conversion of ARA to 15-HETE (15-hydroxyeicosapentaenoic acid), which reduces oxidative damage during follicular development via anti-inflammatory effects, prolonging follicular survival and complementing the role of PLA2G6 in metabolic initiation. Upon stimulation by calcium ionophores, ALOX15B localization increases on both the plasma membrane and the cytoplasmic side of the intracellular membrane [46,47]. Expressed in human macrophages [48], it catalyzes stereospecific peroxidation of polyunsaturated fatty acids (PUFAs) to generate hydroperoxyl derivatives identical to those produced by ALOX15 [49]—supporting its conserved role in lipid signaling. Crucially, this antioxidant activity likely serves as a brake against ferroptosis (iron-dependent cell death), a newly recognized driver of ovarian aging and follicular atresia characterized by lethal lipid peroxidation [50,51,52]. By preventing ferroptosis, ALOX15B extends the functional lifespan of developing follicles, consistent with findings that mitochondrial dysfunction and lipid peroxidation are hallmarks of reproductive senescence [53,53].

*AGPAT3* (lysophosphatidic acid acyltransferase) modulates membrane properties through phospholipid remodeling, helping to maintain a suitable lipid environment for membrane-associated metabolic reactions. During lipid synthesis, AGPAT3 preferentially uses fatty acyl-coenzyme A (formed by ARA/DHA conjugation with coenzyme A) as a substrate, acquiring acyl groups for phospholipid synthesis [54]. It plays a key role in ovarian phospholipid synthesis [55]: AGPAT3-mediated phosphatidic acid synthesis enhances follicular membrane fluidity, providing a crucial structural basis for follicular rupture during ovulation and ensuring ovulation efficiency. Moreover, efficient phospholipid remodeling is a prerequisite for maintaining mitochondria–lipid droplet (LD) contact sites, which are critical hubs for fatty acid transfer and β-oxidation [56]. In HCP hens, the coordinated action of AGPAT3 likely maintains intact LD-Mito contacts, enabling efficient energy production from lipid stores, whereas LCP hens may suffer from contact site disintegration, leading to lipotoxicity and energy failure.

Collectively, these three genes form a complete regulatory chain: substrate supply (PLA2G6) → functional effect (ALOX15B) → environmental support (AGPAT3). Notably, the release of choline-containing phospholipids by PLA2G6 may also fuel cholinergic signaling, where cholinergic depolarization recruits a persistent Ca^2+^ current essential for maintaining long-term cellular excitability and continuous ovulatory cycles [57].

The association confirms that the “intestinal microbiota → serum lipid transfer of → ovarian pathway response” cascade is not a random omics-level correlation but a logically consistent process of material transfer and functional response: gut-derived lipid precursors are transported to the ovary via serum carriers, directly activating core metabolic pathways and providing substrates/structural support for follicular development. Serum-targeted metabolomics validation further strengthens the credibility of the gut–ovarian axis regulation of egg-laying persistence.

**Figure 22:**
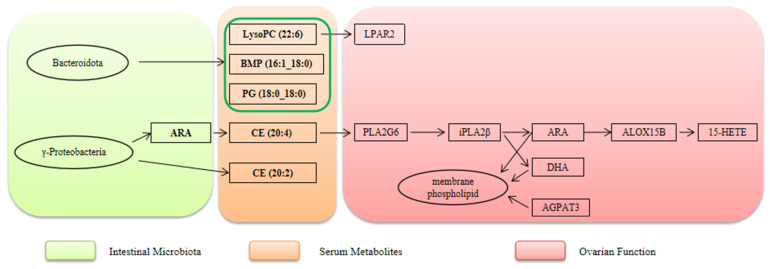
Schematic diagram of the gut–ovarian axis regulated by microbial-derived lipid mediators. Note: The arrows indicate the direction of biological effects or metabolic flow. The green box highlights three key serum metabolites (LysoPC, BMP, and PG) identified as important mediators in this pathway.

HCP and LCP serum-targeted metabolomics data provide critical experimental validation of the proposed “gut–ovary axis” from two distinct yet interconnected aspects (Figure 22): (1) by identifying specific lipid carriers (e.g., CE (20:4)) that constitute a molecular bridge for gut-derived signal transmission to the systemic circulation, and (2) by demonstrating the differential abundance of key lipid precursors that serve as direct substrates for the activated ovarian lipid metabolic pathway. This evidence robustly corroborates the “gut–ovary axis lipid mediator regulatory model” inferred from the preceding multi-omics integration. Collectively, the data support a mechanistic narrative wherein the HCP phenotype is characterized by an enriched intestinal microbiota that synthesizes specific lipid precursors. These precursors are efficiently transported via the serum to the ovary, where they contribute to activating the core PLA2G6-ALOX15B-AGPAT3 axis and its regulated phospholipid metabolic network, thereby creating a metabolic milieu conducive to sustained follicular development and high clutch persistence. In contrast, the LCP group exhibits a profile of serum lipid dysregulation. This altered lipid profile is associated with an attenuated transmission of intestinal signals and an inadequate supply of substrates to the ovarian lipid pathways, culminating in compromised follicular development and reduced egg-laying persistence. These findings refine the mechanistic understanding of the gut–ovary axis and establish a theoretical foundation for potential interventions—such as modulation of intestinal microbiota or systemic lipid metabolism—aimed at improving laying performance in aged hens. Future studies may leverage these insights to develop targeted strategies, for instance, by intervening with key microbial taxa or lipid mediators like LysoPC (22:6), to enhance clutch persistence [58].

A notable limitation of this study is the modest sample size for the in-depth omics analyses, a trade-off necessitated by their high cost. We mitigated this limitation by applying strict statistical cutoffs and, more importantly, by seeking convergent signals across independent omics datasets. Nonetheless, the generalizability of the specific molecular features identified here requires confirmation in larger populations. The primary contribution of this work is the establishment of a novel, systemic “gut–serum–ovary” framework for understanding clutch persistence. Future research should prioritize validating this model in expanded cohorts and testing the causal role of its key components through direct dietary or microbial interventions.

## 5. Conclusions

This study investigated the regulatory mechanisms underlying clutch persistence in 65-week-old laying hens using multi-omics analyses, proposing a lipid mediator network-mediated “gut–ovarian axis” model that extends the traditional “ovary-centric” paradigm. Key findings identified distinct profiles of cecal microbiota, serum metabolites, and ovarian regulatory pathways associated with high clutch persistence. Specifically, HCP-enriched γ-Proteobacteria drove lipid precursor biosynthesis, which optimized the follicular microenvironment—potentially via downregulating ovarian TNF-α. Serum LysoPC (22:6), a critical signaling molecule identified herein, activated ovarian LPAR2 to the continuity of follicular development. The ovarian PLA2G6-ALOX15B-AGPAT3 axis orchestrated lipid metabolism, directly supporting follicle maturation and ovulation. Collectively, these results demonstrate that gut microbiota-derived lipid mediators bridge intestinal and ovarian function, forming a “microbiota–serum–ovary” regulatory cascade. This work provides novel insights for extending the economic laying cycle of aged hens and identifies promising targets to enhance poultry production efficiency.

## Figures and Tables

**Table 1:** Composition and calculated nutrient levels of the experimental diet (as-fed basis).

Ingredient	Content (%)	Nutrient	Content/Level
Corn	61.4	Metabolizable energy (MJ/kg)	11.5
Soybean meal	21	Crude protein (%)	16.5
Wheat bran	14	Calcium (%)	3.5
Dicalcium phosphate	1.2	Available phosphorus (%)	0.42
Limestone	1.1	Crude fat (%)	3.2
Salt	0.3		
Vitamin-mineral premix 1	1		
Total	100.0		

Note: The premix provided per kilogram of diet: Vitamin A, 12,500 IU; Vitamin D_3_, 4125 IU; Vitamin E, 30 IU; Vitamin K_3_, 2 mg; Thiamine, 2 mg; Riboflavin, 8 mg; Niacin, 50 mg; Pantothenic acid, 15 mg; Pyridoxine, 5 mg; Biotin, 0.2 mg; Folic acid, 1 mg; Vitamin B_12_, 0.02 mg; Choline chloride, 500 mg; Fe (as ferrous sulfate), 80 mg; Cu (as copper sulfate), 8 mg; Mn (as manganese sulfate), 100 mg; Zn (as zinc sulfate), 80 mg; I (as potassium iodide), 1 mg; Se (as sodium selenite), 0.3 mg.

**Table 2:** Primer sequences for qRT-PCR.

Gene	Accession Number (NCBI)	Primer Sequence (5′→3′)	Product Size (bp)
PLA2G6	XM_015285287.4	Forward: GTGCCATGCAGTTCCTC	200
		Reverse: GTGGAAGAGCCGGAAG	
ALOX15B	XM_021067854.1	Forward: AATCTGGAGCTATGGAAGGACG	195
		Reverse: GATAGATGCTCCGAGGGTGG	
AGPAT3	XM_040659767.1	Forward: TCCAGACGTCTTGGCTCAAC	123
		Reverse: GGAACGTTGCCAATGCTGTT	
β-actin	NM_205518.2	Forward: GGCCCATACCAACCATCACA	112
		Reverse: AATGGCTCCGGTATGTGCAA	

**Table 3:** Ovarian metabolomic data preprocessing results.

Ion Mode	Effective Peak (Raw)	Identified Metabolites (Raw)	Proportion (%)	Effective Peak (Origin)	Identified Metabolites (Origin)	Proportion (%)
Pos	4454	542	12.17%	3131	479	15.3%
Neg	4296	272	6.33%	3336	246	7.37%
mix	8750	814	9.3%	6467	725	11.21%

Note: “raw” denotes the original unprocessed data table; “origin” refers to the data table derived from preprocessing of the raw table. Preprocessing parameters are available for review in the preprocessing interface. The origin table was used for all subsequent analyses.

**Table 4:** Total ion count and metabolite identification statistics of ovarian metabolome.

Ion Mode	All Peaks	Identified Metabolites	Unidentified	Metabolites in Library	Metabolites in KEGG
Pos	3131	479	2652	381	202
Neg	3336	246	3090	211	126
mix	6467	725	5742	592	328

Note: (1) Ion mode: Ionization mode of analytes detected by mass spectrometry (MS), mainly including Pos and Neg; (2) Total peaks: Number of MS peaks extracted by data processing software; (3) Identified metabolites: Number of metabolites finally identified via MS^1^/MS^2^ data and library searching; (4) Library-annotated metabolites: Number of metabolites annotated in public databases; (5) KEGG-annotated metabolites: Number of metabolites annotated in the KEGG database.

## Data Availability

The data presented in this study are available upon request from the corresponding author. The data will be made publicly available in a reputable repository upon acceptance of the manuscript.
